# Metabolomic Analysis of the Skeletal Muscle of Mice Overexpressing PGC-1α

**DOI:** 10.1371/journal.pone.0129084

**Published:** 2015-06-26

**Authors:** Yukino Hatazawa, Nanami Senoo, Miki Tadaishi, Yoshihiro Ogawa, Osamu Ezaki, Yasutomi Kamei, Shinji Miura

**Affiliations:** 1 Department of Molecular Endocrinology and Metabolism, Graduate School of Medical and Dental Sciences, Tokyo Medical and Dental University, Tokyo, Japan; 2 Laboratory of Molecular Nutrition, Graduate School of Environmental and Life Science, Kyoto Prefectural University, Kyoto, Japan; 3 Laboratory of Nutritional Biochemistry, Graduate School of Nutritional and Environmental Sciences, University of Shizuoka, Shizuoka, Japan; 4 Department of Nutritional Science, Faculty of Applied Bio-Science, Tokyo University of Agriculture, Tokyo, Japan; 5 Department of Human Health and Design, Showa Women’s University, Tokyo, Japan; University of Debrecen, HUNGARY

## Abstract

Peroxisome proliferator-activated receptor (PPAR) γ coactivator 1α (PGC-1α) is a coactivator of various nuclear receptors and other transcription factors whose expression increases in the skeletal muscle during exercise. We have previously made transgenic mice overexpressing PGC-1α in the skeletal muscle (PGC-1α-Tg mice). PGC-1α upregulates the expression of genes associated with red fibers, mitochondrial function, fatty acid oxidation, and branched chain amino acid (BCAA) degradation. However, global analyses of the actual metabolic products have not been investigated. In this study, we conducted metabolomic analysis of the skeletal muscle in PGC-1α-Tg mice by capillary electrophoresis with electrospray ionization time-of-flight mass spectrometry. Principal component analysis and hierarchical cluster analysis showed clearly distinguishable changes in the metabolites between PGC-1α-Tg and wild-type control mice. Changes were observed in metabolite levels of various metabolic pathways such as the TCA cycle, pentose phosphate pathway, nucleotide synthesis, purine nucleotide cycle, and amino acid metabolism, including BCAA and β-alanine. Namely, metabolic products of the TCA cycle increased in PGC-1α-Tg mice, with increased levels of citrate (2.3-fold), succinate (2.2-fold), fumarate (2.8-fold), and malate (2.3-fold) observed. Metabolic products associated with the pentose phosphate pathway and nucleotide biosynthesis also increased in PGC-1α-Tg mice. Meanwhile, BCAA levels decreased (Val, 0.7-fold; Leu, 0.8-fold; and Ile, 0.7-fold), and Glu (3.1-fold) and Asp (2.2-fold) levels increased. Levels of β-alanine and related metabolites were markedly decreased in PGC-1α-Tg mice. Coordinated regulation of the TCA cycle and amino acid metabolism, including BCAA, suggests that PGC-1α plays important roles in energy metabolism. Moreover, our metabolomics data showing the activation of the purine nucleotide pathway, malate–aspartate shuttle, as well as creatine metabolism, which are known to be active during exercise, further suggests that PGC-1α regulates metabolism in exercise. Thus, we demonstrated the roles of PGC-1α in the skeletal muscle at the metabolite level.

## Introduction

Peroxisome proliferator-activated receptor (PPAR) γ coactivator 1α (PGC-1α) is a coactivator of various nuclear receptors and other transcription factors, which is involved in the regulation of energy metabolism, thermogenesis, and other biological processes that control phenotypic characteristics of various organ systems, including the skeletal muscle [1–5]. PGC-1α in the skeletal muscle is thought to be involved in contractile protein function, mitochondrial function, metabolic regulation, intracellular signaling, and transcriptional responses, and its levels increase in the skeletal muscle with exercise.

Animal and cellular genetic models with altered expression of the PGC-1α gene indicate the role of PGC-1α in fiber-type specificity [6, 7]. We have previously demonstrated that transgenic overexpression of PGC-1α in the skeletal muscle of mice (PGC-1α-Tg mice) increases mitochondrial biogenesis and capillary density, contributing to improved exercise capacity [4]. Meanwhile, in a previous study, a microarray analysis revealed that the BCAA catabolic pathway was coordinately activated in the skeletal muscle of PGC-1α-Tg mice. It was apparent that PGC-1α stimulates the metabolism of branched chain amino acids (BCAA) with an increase in the expression of enzymes involved [8].

Considering that PGC-1α changes the expression of various genes in the skeletal muscle, including those involved in muscle metabolism, metabolites are also expected to change; however, little is known about the global changes in metabolites in PGC-1α-Tg mice. Global information of metabolite level change may reveal connections in the biological network of the skeletal muscle in these mice. Thus, we analyzed metabolic profiles by coupling capillary electrophoresis with electrospray ionization time-of-flight mass spectrometry (CE-TOFMS). In the present study, combined with microarray data [8], we analyzed global changes of metabolites in the skeletal muscle of PGC-1α-Tg mice to investigate the modified metabolic pathways related to PGC-1α expression.

## Results and Discussion

**Fig 1:**
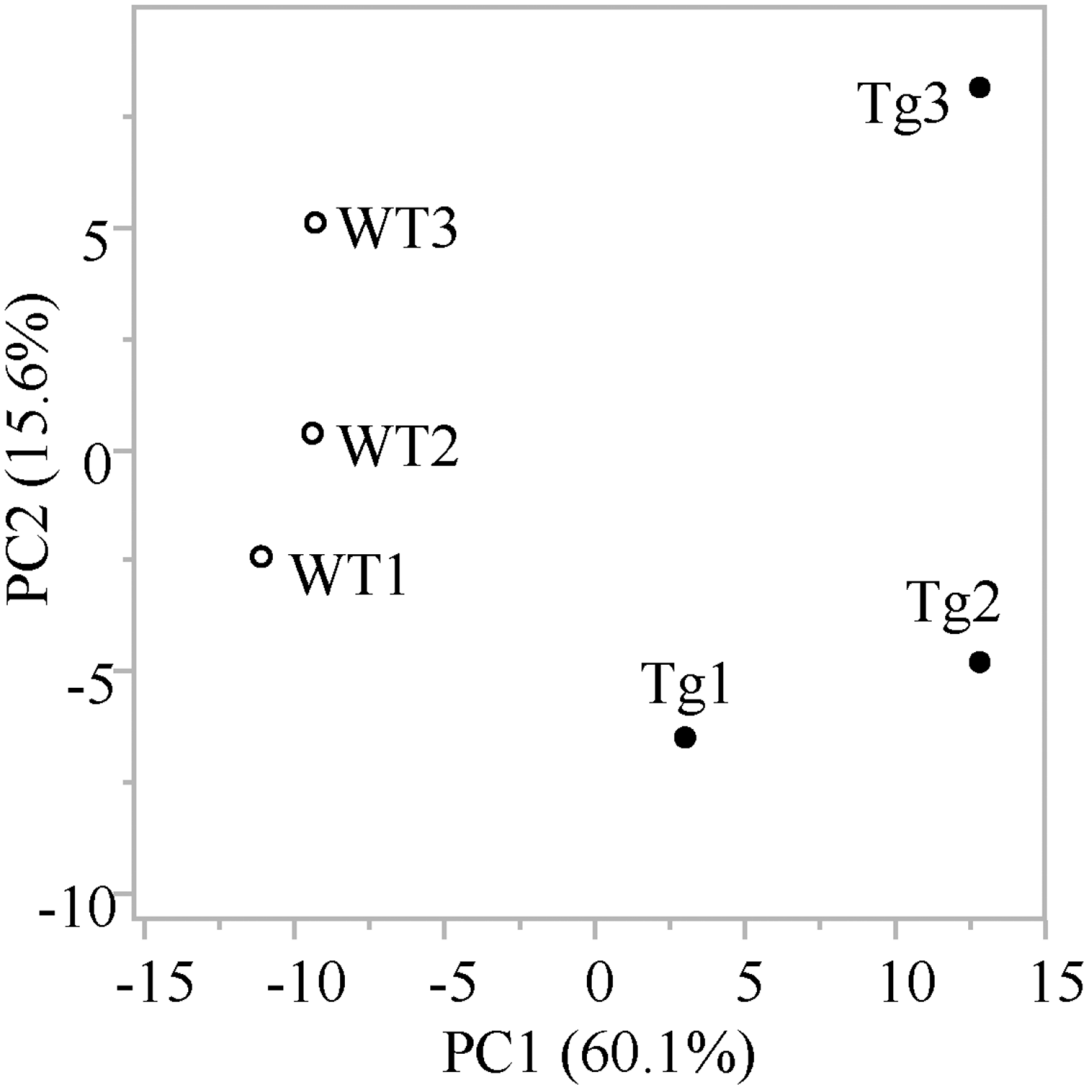
Principal component analysis (PCA) of the metabolomic datasets of the skeletal muscle of PGC-1α-Tg mice and WT mice. Three mice were used in each group (WT1, WT2, and WT3 for wild-type and Tg1, Tg2, and Tg3 for PGC-1α-Tg mice). PCA was conducted with the determined data peaks by using SampleStat ver. 3.14. Plots of WT (open circles) and PGC-1α-Tg mice (filled circles) are clearly distinguished on the PC1 axis (X-axis).

**Fig 2:**
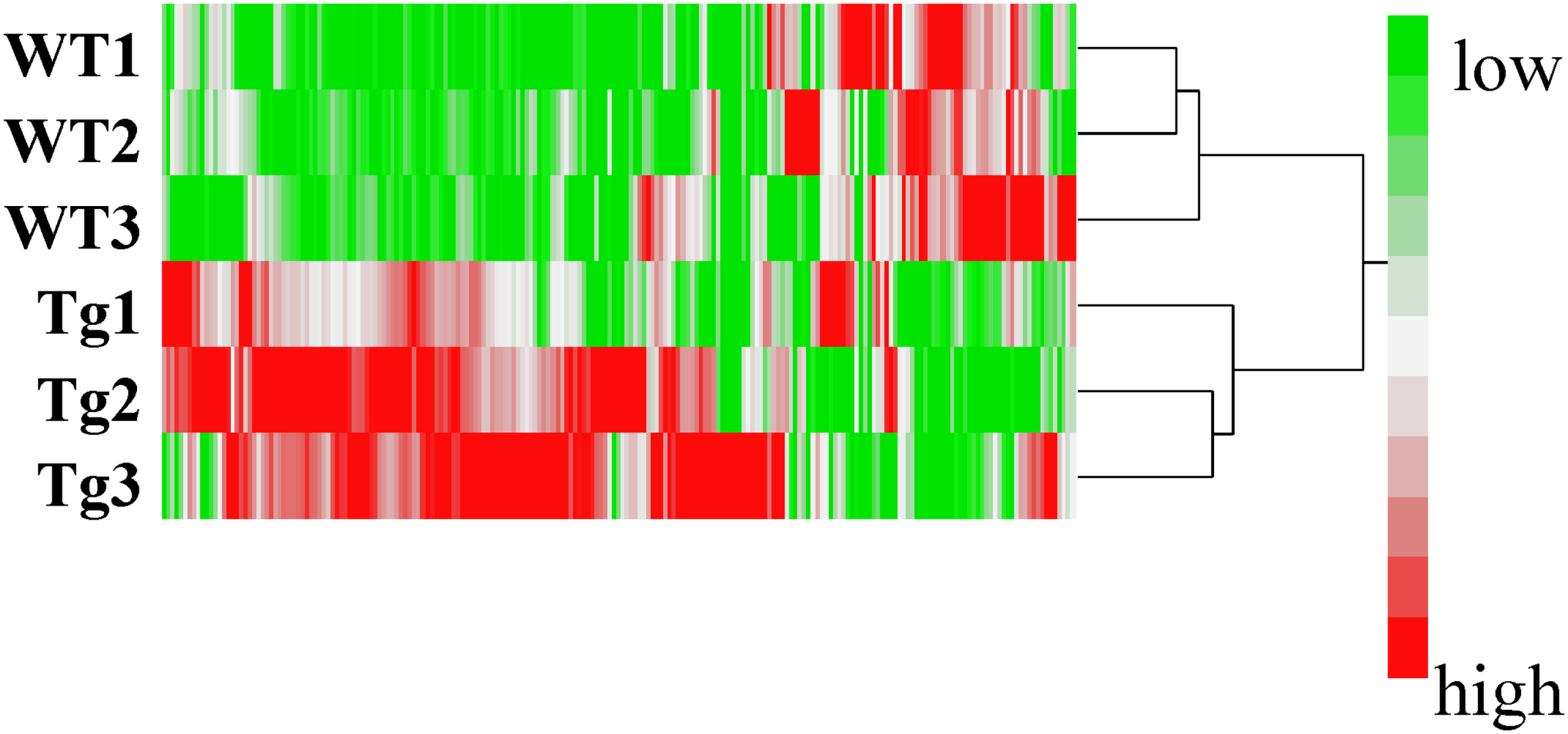
A heat map of hierarchical cluster analysis comparing the metabolite changes between PGC-1α-Tg mice and WT mice. Horizontal axis shows sample names corresponding to the samples used in Fig 1 (WT1, WT2, and WT3 for wild-type and Tg1, Tg2, and Tg3 for PGC-1α-Tg mice). The heat map patterns between WT (upper three lanes) and PGC-1α-Tg (lower three lanes) are clearly distinguishable. The color red demonstrates that the relative content of metabolites is high and green demonstrates that they are low.

Metabolomic analysis was conducted in the skeletal muscle of PGC-1α-Tg mice with age- and sex-matched wild-type (WT) mice littermates. Average body weights were 26.2 ± 3.0 g in PGC-1α-Tg mice and 25.7 ± 2.0 g in WT mice. Average weights of the gastrocnemius muscles were 115 ± 10 mg in PGC-1α-Tg mice and 138 ± 17 mg in WT mice. Consistent with previous reports [4], the weights of the gastrocnemius muscles in PGC-1α-Tg mice were significantly lower than those in WT littermates. Skeletal muscles of PGC-1α-Tg mice showed a red color characteristic of oxidative muscle. In the metabolomic analysis, 211 peaks (126 cations and 85 anions) were detected by the anion and cation modes of CE-TOFMS. The results of principal component analysis (PCA) in these detected peaks are shown in Fig 1. The first principal component effectively and distinctly separated the mice based on genotype (x axis), suggesting that overexpression of PGC-1α in the skeletal muscle caused a significant change in the overall metabolite profile of the muscle. Furthermore, a hierarchical cluster analyses (HCA) was conducted, followed by heat map analysis (Fig 2). As demonstrated from the heat map analysis, skeletal muscle samples from individual WT and PGC-1α-Tg mice segregated into tight clusters, indicating that PGC-1α has profound effects on the systemic metabolite profile of the skeletal muscle. From the results of PCA (Fig 1) and HCA (Fig 2), it was observed that PGC-1α overexpression had a significant influence in the metabolite profiles of the skeletal muscle because the two groups (WT and PGC-1α-Tg) were clearly distinguishable. The relative area values of the detected metabolic products in PGC-1α-Tg mice and WT are listed in S1 Table, sorted in order (PGC-1α-Tg per WT). In the following subsections, we discuss the results of the metabolomic analysis.

### TCA cycle

**Fig 3:**
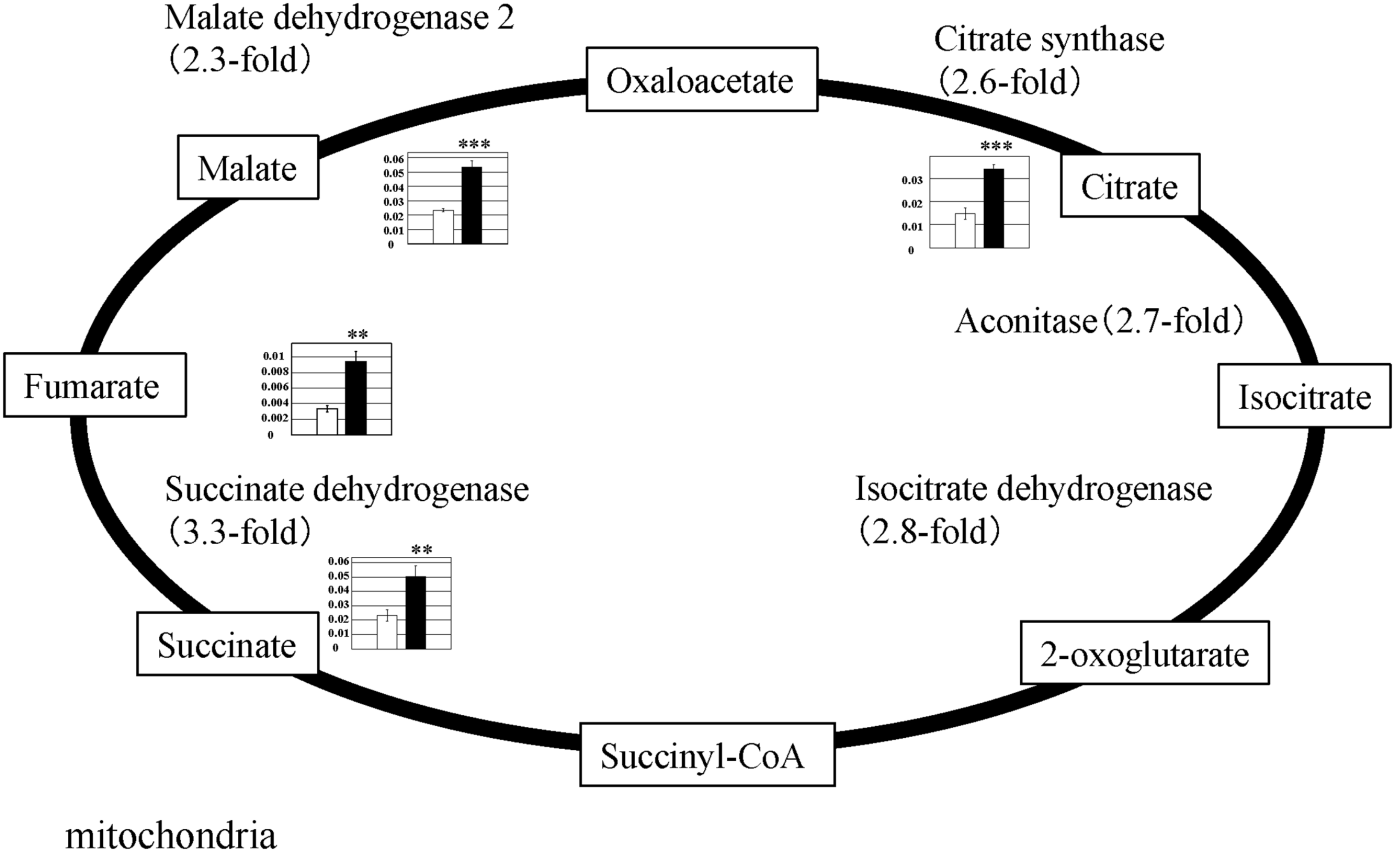
Observed metabolite changes mapped onto the pathways involved in the TCA cycle. Changes in the metabolite levels in the skeletal muscle of PGC-1α-Tg mice and WT mice are shown. Relative metabolite changes shown in the graphs were obtained by CE-TOFMS (S1 Table). Open bars, WT and filled bars, PGC-1α-Tg (N = 3). Data are expressed as the mean ± SD. Asterisks indicate statistically significant differences (***p < 0.001, **p < 0.01). Microarray data of gene expression change of enzymes in the related metabolic process are shown in the scheme.

Metabolic products of the TCA cycle increased in PGC-1α-Tg mice. The levels of citrate (2.3-fold), succinate (2.2-fold), fumarate (2.8-fold), and malate (2.3-fold) increased (Fig 3 and S1 Table). Consistent with the increased metabolic product levels of the TCA cycle, the gene expression of citrate synthase (2.6-fold), aconitase (2.7-fold), isocitrate dehydrogenase (2.8-fold), succinate dehydrogenase (3.3-fold), and malate dehydrogenase 2 (2.3-fold) increased in PGC-1α-Tg mice (Fig 3). These data suggest that the TCA cycle was activated in PGC-1α-Tg mice, probably, in part, due to the increase of mitochondria content [4].

### Pentose phosphate pathway

**Fig 4:**
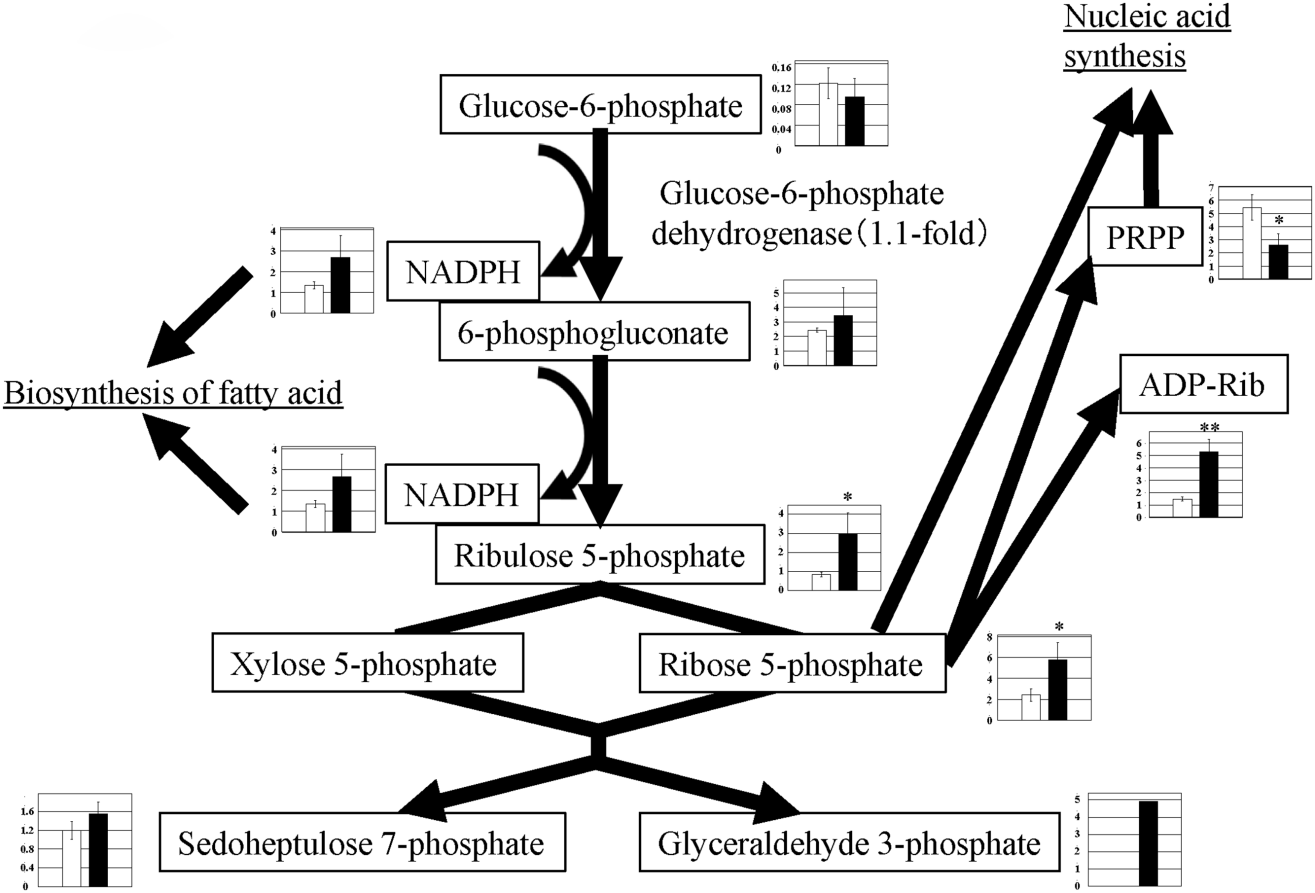
Observed metabolite changes mapped onto the pathways involved in the pentose phosphate pathway. Changes in the metabolite levels in the skeletal muscle of PGC-1α-Tg mice and WT mice are shown. Relative metabolite changes shown in the graphs were obtained by CE-TOFMS (S1 Table). Open bars, WT and filled bars, PGC-1α-Tg (N = 3). Data are expressed as the mean ± SD. Asterisks indicate statistically significant differences (**p < 0.01, *p < 0.05). Microarray data of gene expression change of enzymes in the related metabolic process are shown in the scheme.

The pentose phosphate pathway is initiated when glucose 6-phosphate, a metabolic intermediate of glycolysis, is metabolized to 6-phosphogluconate. The pentose phosphate pathway produces ribose 5-phosphate that is required for nucleotide biosynthesis. In addition, the pathway produces NADPH, a reducing agent required for de novo lipogenesis (Fig 4 and S1 Table) [9, 10]. In this study, metabolic products associated with the pentose phosphate pathway increased in PGC-1α-Tg mice; the levels of 6-phosphogluconate (1.4-fold), ribulose 5-phosphate (3.6-fold), ribose 5-phosphate (2.4-fold), NADPH (2.0-fold), ADP-ribose (3.6-fold), and sedoheptulose 7-phosphate (1.3-fold) increased in PGC-1α-Tg mice compared with WT mice (Fig 4 and S1 Table). Glyceraldehyde 3-phosphate was not detected in WT mice, but detected in one mouse among three PGC-1α-Tg mice. However, the gene expression of enzymes associated with this pathway was not changed, consistent with previous reports that PGC-1α promoted the pentose phosphate pathway by enzymatic activity (glucose 6-phosphate dehydrogenase), but not gene expression [11]. Thus, PGC-1α-Tg mice in this study are likely to have a more active pentose phosphate pathway, as previously reported [11]. As suggested in this previous report, an increase in NADPH content observed in our PGC-1α-Tg mice may contribute to the stimulation of lipogenesis in the skeletal muscle.

### Nucleotide biosynthesis

**Fig 5:**
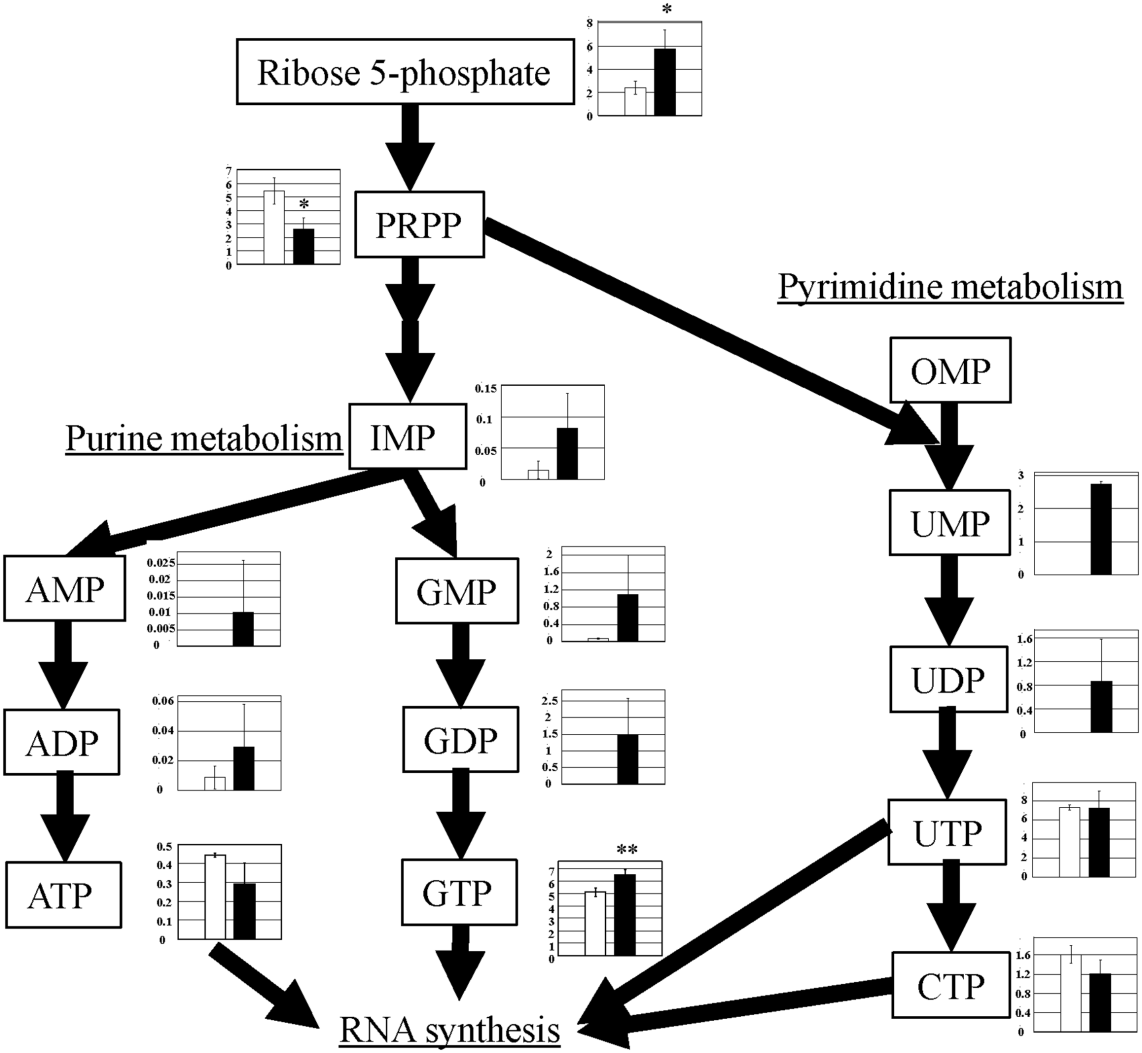
Observed metabolite changes mapped onto the pathways involved in nucleotide synthesis. Changes in the metabolite levels in the skeletal muscle of PGC-1α-Tg mice and WT mice are shown. Relative metabolite changes shown in the graphs were obtained by CE-TOFMS (S1 Table). Open bars, WT and filled bars, PGC-1α-Tg (N = 3). Data are expressed as the mean ± SD. Asterisks indicate statistically significant differences (**p < 0.01, *p < 0.05).

Ribose 5-phosphate, derived from the pentose phosphate pathway, is a starting material for nucleotide biosynthesis, including pyrimidines and purines [9, 10]. We observed a change in metabolite levels of these pathways. For pyrimidine biosynthesis, ribose 5-phosphate (2.4-fold) is metabolized to 5-phosphoribosyl pyrophosphate (PRPP) (0.5-fold), uridine monophosphate (UMP) (detected only in PGC-1α-Tg mice, but not in WT mice), uridine diphosphate (UDP) (detected only in PGC-1α-Tg mice, but not in WT mice), uridine triphosphate (UTP) (1.0-fold), and cytidine triphosphate (CTP) (0.7-fold) (Fig 5 and S1 Table). For purine biosynthesis, ribose 5-phosphate (2.4-fold) is metabolized to inosine monophosphate (IMP) (5.4-fold), which is used in the purine nucleotide cycle (described in the next paragraph). IMP is metabolized to AMP (detected only in PGC-1α-Tg mice, but not in WT control mice), ADP (3.4-fold), and ATP (0.7-fold). IMP is also metabolized to GMP (16-fold), GDP (detected only in PGC-1α-Tg mice, but not in WT mice), and GTP (1.3-fold) (Fig 5 and S1 Table). Among them, UTP, CTP, ATP, and GTP may be used for RNA synthesis, which is consistent with the previous report that RNA synthesis is stimulated in PGC-1α-Tg mice [11]. Because many of the associated metabolites are increased, the nucleotide biosynthesis pathway appears to be activated in PGC-1α-Tg mice.

### Purine nucleotide cycle

**Fig 6:**
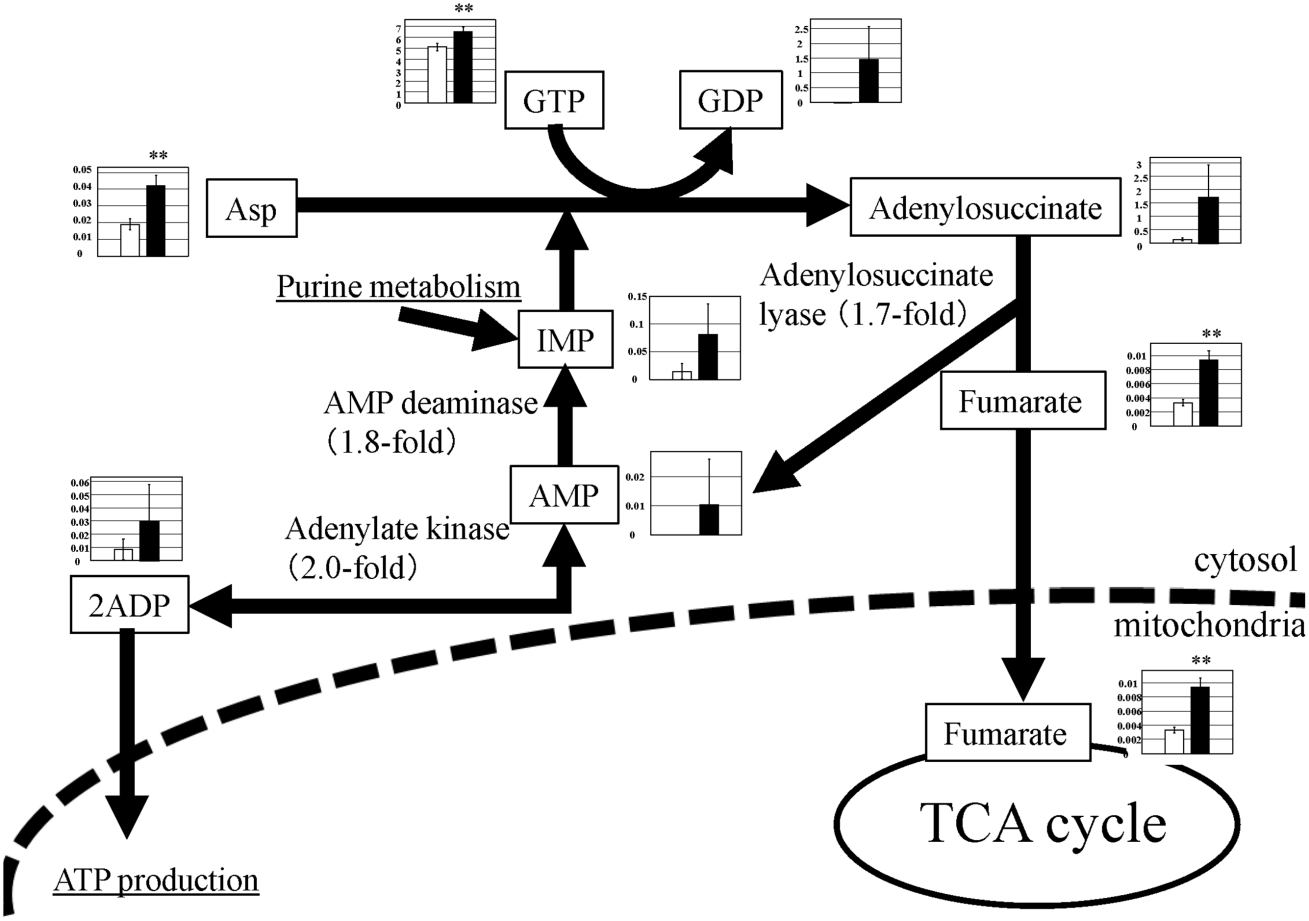
Observed metabolite changes mapped onto the pathways involved in the purine nucleotide cycle. Changes in the metabolite levels in the skeletal muscle of PGC-1α-Tg mice and WT mice are shown. Relative metabolite changes shown in the graphs were obtained by CE-TOFMS (S1 Table). Open bars, WT and filled bars, PGC-1α-Tg (N = 3). Data are expressed as the mean ± SD. Asterisks indicate statistically significant differences (**p < 0.01). Microarray data of gene expression change of enzymes and transporter in the related metabolic process are shown in the scheme.

The purine nucleotide cycle is a metabolic pathway that contributes to the energy requirement in the skeletal muscle, and is very active during exercise [12]. An outline of the purine nucleotide cycle is shown in Fig 6.

In the activated purine nucleotide cycle, fumarate is supplied to the TCA cycle, enhancing the capacity of acetyl-CoA oxidation. As shown in Fig 6, adenylosuccinate is the product of Asp and IMP (generated from AMP), which is then metabolized into fumarate. Metabolic product levels related to this pathway were changed in PGC-1α-Tg mice. The levels of GDP and AMP were detected in PGC-1α-Tg mice but not in WT mice (Fig 6 and S1 Table). Consistently, increasing AMP levels were observed in a previous study using a different line of PGC-1α-Tg mice [13]. Meanwhile, in the metabolome data, adenylosuccinate (12-fold), fumarate (2.8-fold), IMP (5.4-fold), Asp (2.2-fold), GTP (1.3-fold), and ADP (3.4-fold) levels increased in PGC-1α-Tg mice (Fig 6 and S1 Table). Microarray data show an increase in related enzyme gene expression: AMP deaminase (AMP → IMP) (1.8-fold), adenylosuccinate lyase (adenylosuccinate → fumarate + AMP) (1.7-fold), and adenylate kinase (AMP + ATP ←→ 2ADP) (2-fold). Thus, the purine nucleotide cycle appears to be activated in PGC-1α-Tg mice. This is consistent with increased activity of the pentose phosphate pathway and nucleotide synthesis, as the metabolites derived from those pathways, such as IMP, are used for purine nucleotide cycle. Also, this is consistent with an increased mitochondria level in PGC-1α-Tg mice [4], as ADP derived from purine nucleotide cycle is usable for ATP production in the mitochondria.

### BCAA metabolism and malate-aspartate shuttle

**Fig 7:**
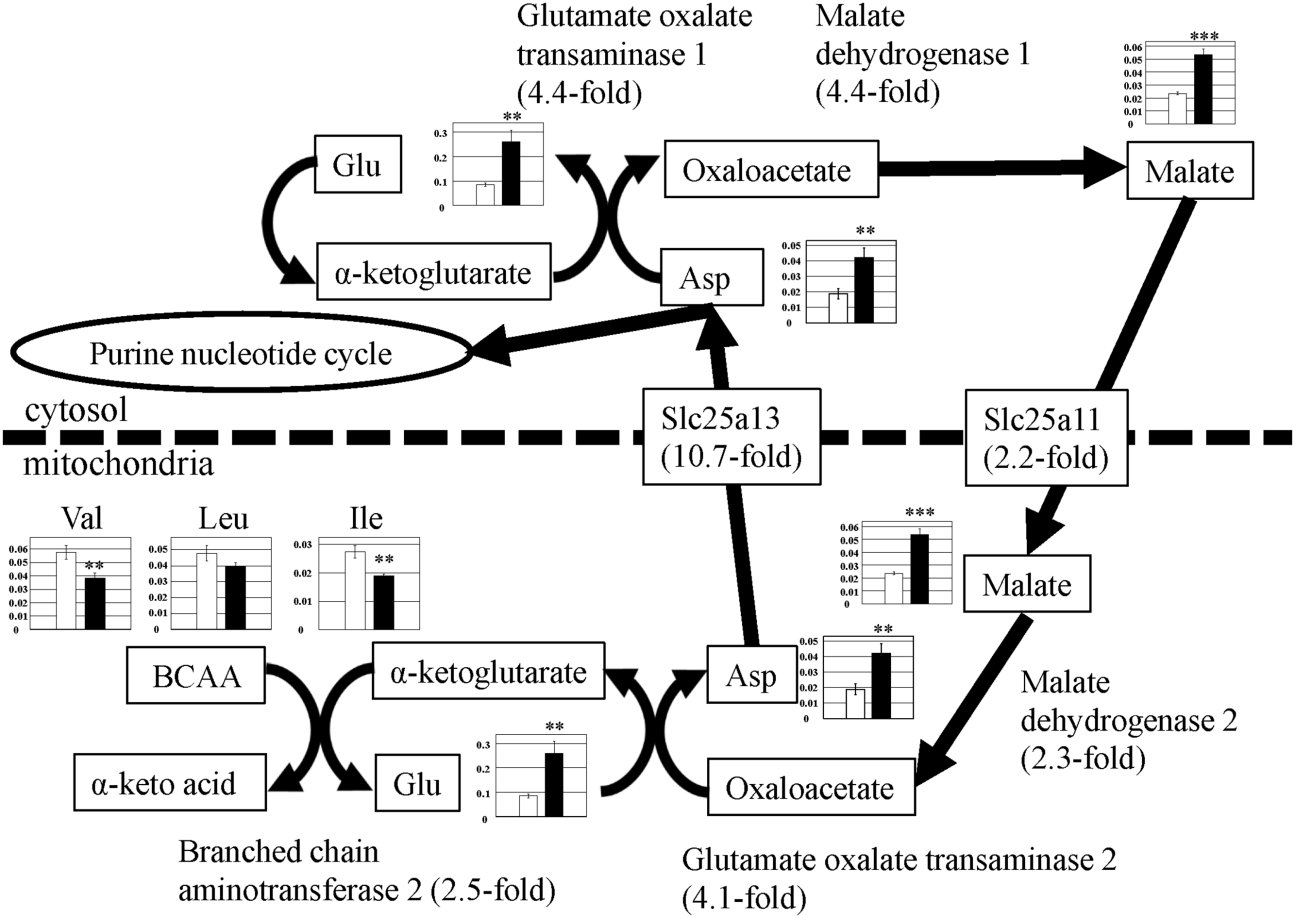
Observed metabolite changes mapped onto the pathways associated with BCAA metabolism and the malate-aspartate shuttle. Changes in the metabolite levels in the skeletal muscle of PGC-1α-Tg mice and WT mice are shown. Relative metabolite changes shown in the graphs were obtained by CE-TOFMS (S1 Table). Open bars, WT and filled bars, PGC-1α-Tg (N = 3). Data are expressed as the mean ± SD. Asterisks indicate statistically significant differences (***p < 0.001, **p < 0.01). Microarray data of gene expression change of enzymes and transporter in the related metabolic process are shown in the scheme.

We have previously reported that BCAA metabolism was enhanced in PGC-1α-Tg mice [8]; in accordance, levels of BCAA (Val, Leu, and Ile) decreased in the present metabolomic analysis (Fig 7 and S1 Table) (Val, 0.7-fold; Leu, 0.8-fold; and Ile, 0.7-fold). Leu and Ile are degraded, producing acetyl-CoA, and enters the TCA cycle [9, 10]. The level of acetyl-CoA was more increased in PGC-1α-Tg mice than in WT mice (detected only in PGC-1α-Tg mice, and not in WT mice, S1 Table). Val and Ile are converted into succinyl-CoA and enters the TCA cycle [9, 10]. Thus, this metabolomic analysis further supports our previous study of increased BCAA metabolism, which is likely to be used in the TCA cycle [8]. Meanwhile, Val is known to be converted into methylmalonic acid semialdehyde, followed by the production of β-amino isobutyric acid (BAIBA). BAIBA was detected only in PGC-1α-Tg mice, but not in WT mice (S1 Table), consistent with previous reports that the level of BAIBA increased in cells overexpressing PGC-1α [14].

On the other hand, the increased amino acids in PGC-1α-Tg mice in the metabolomic analysis were as follows: Glu (3.1-fold), Asp (2.2-fold), Arg (1.7-fold), Gln (1.7-fold), and Lys (1.6-fold) (S1 Table). As shown in Fig 7, in the mitochondria, Asp is converted from oxaloacetate and is then transported from the mitochondria to the cytosol via the Slc25a13 transporter. Asp in the cytosol is then converted to oxaloacetate and malate. Furthermore, malate is transported from the cytosol to the mitochondria via Slc25a11 [15] and is converted to oxaloacetate, which is a part of the TCA cycle [9, 10]. This metabolic pathway is known as the malate–aspartate shuttle (known to be associated with BCAA metabolism during exercise) [16]. The gene expression of enzymes and transporters involved in this process were upregulated in PGC-1α-Tg mice; glutamate oxalate transaminase (GOT2) (catalyzes oxaloacetate to Asp in mitochondria) was upregulated by 4.1-fold, Slc25a13 (a transporter of Asp) [15] by 10.7-fold, GOT1 (Asp to oxaloacetate in cytosol) by 4.4 fold, malate dehydrogenase 1 (oxaloacetate to malate in cytosol) by 4.4-fold, Slc25a11 (a transporter of malate) [15] by 2.2-fold, and malate dehydrogenase 2 (malate to oxaloacetate in mitochondria) by 2.3-fold. Concerning mitochondrial Asp, GOT2 metabolization of Asp results in Glu conversion to α-keto-glutarate. Meanwhile, BCAA is converted to α-keto-acid, which is catalyzed by branched chain aminotransferase 2 [9, 10] (2.5-fold upregulated). Consistently, BCAA levels decreased as previously mentioned (Val, 0.7-fold; Leu, 0.8-fold; and Ile, 0.7-fold), and Glu (3.1-fold) levels increased (Fig 7 and S1 Table). Thus, BCAA and related amino acid metabolism as well as the malate-aspartate shuttle appear coordinately regulated in PGC-1α-Tg mice. These results suggest that deamination was promoted with an increase in BCAA metabolism and the activation of the malate-aspartate shuttle, which may contribute to the improvement of exercise capacity of PGC-1α-Tg mice.

### Metabolism of other amino acids with decreased levels

**Fig 8:**
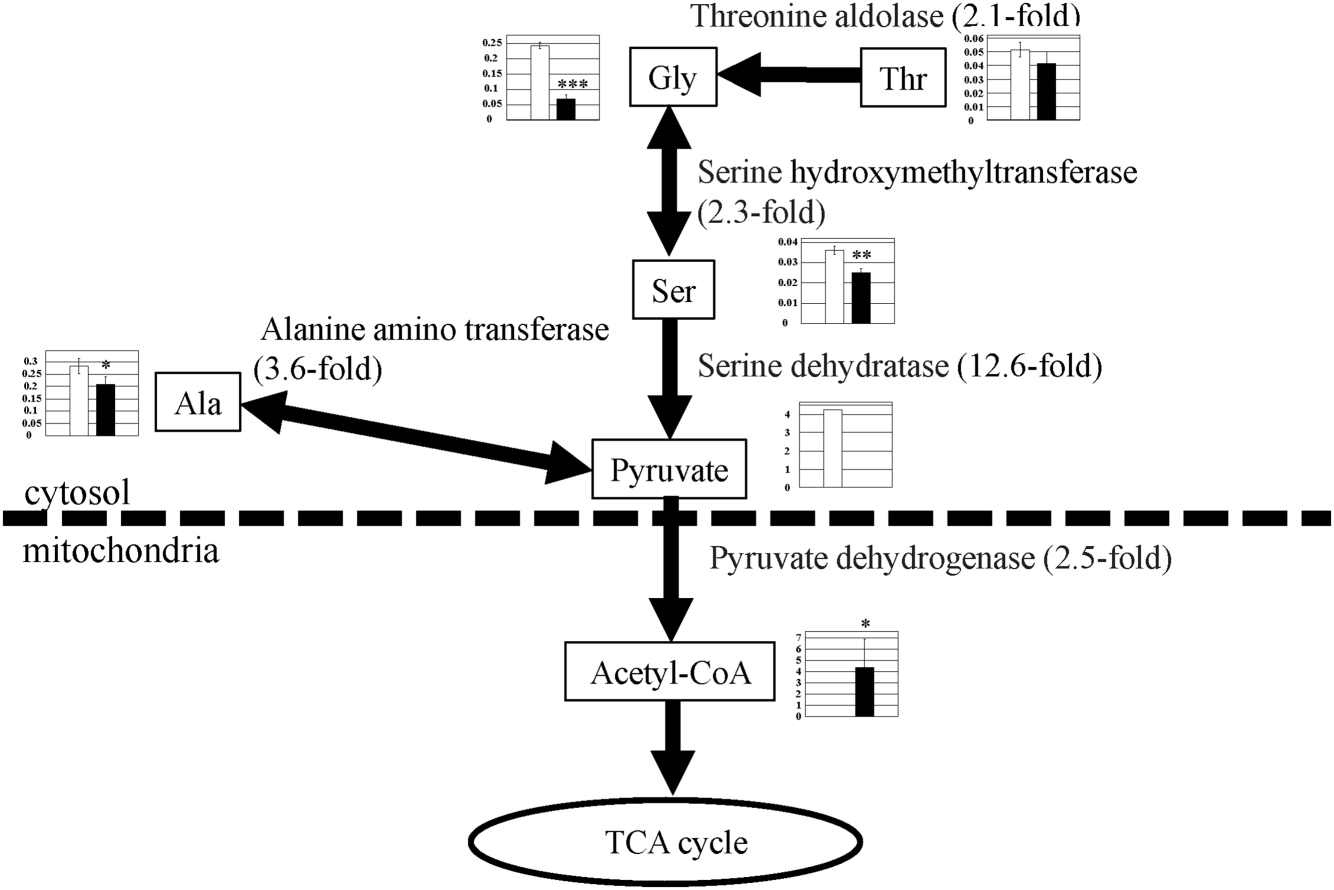
Observed metabolite changes mapped onto the pathways associated with glycine, threonine, serine, and alanine metabolism. Changes in the metabolite levels in the skeletal muscle of PGC-1α-Tg mice and WT mice are shown. Relative metabolite changes shown in the graphs were obtained by CE-TOFMS (S1 Table). Open bars, WT and filled bars, PGC-1α-Tg (N = 3). Data are expressed as the mean ± SD. Asterisks indicate statistically significant differences (***p < 0.001, **p < 0.01, *p < 0.05). Microarray data of gene expression change of enzymes and transporter in the related metabolic process are shown in the scheme.

Some amino acid levels decreased, including Thr (0.7-fold), Met (0.8-fold), Ala (0.7-fold), Ser (0.7-fold), Pro (0.4-fold), and Gly (0.3-fold) in PGC-1α-Tg mice compared with WT mice (S1 Table). Some of these can be converted into pyruvate [9, 10] (Fig 8). Ala is metabolized into pyruvate by alanine amino transferase (ALT). Thr is converted into Gly by threonine aldolase, Gly is subsequently converted into Ser by serine hydroxymethyltransferase, and Ser is subsequently converted into pyruvate by serine dehydratase [9, 10] (Fig 8). The enzymes related to the metabolism of these amino acids increased in this study. Expression of ALT (3.6-fold), threonine aldolase (2.1-fold), serine hydroxymethyltransferase (2.3-fold), and serine dehydratase (12.6-fold) increased. Pyruvate may be converted into acetyl-CoA by pyruvate dehydrogenase (2.5-fold) (Fig 8). Consistently, although there is an increased level of pyruvate dehydrogenase kinase 4, which suppresses pyruvate dehydrogenase activity, pyruvate dehydrogenase activity is enhanced in PGC-1α-Tg mice [17]. In the previous study, we reported that glycolysis was suppressed in PGC-1α-Tg mice, and the respiration quotient was low [4], suggesting that glucose was not used as an energy source. Meanwhile, as the respiration quotient due to the use of amino acids as an energy source is lower than that of glucose, the idea that these amino acids are used for the TCA cycle, via pyruvate and acetyl-CoA, does not contradict with previous findings (suppressed glycolysis and low respiration quotient in PGC-1α-Tg mice) [4].

### β-alanine

**Fig 9:**
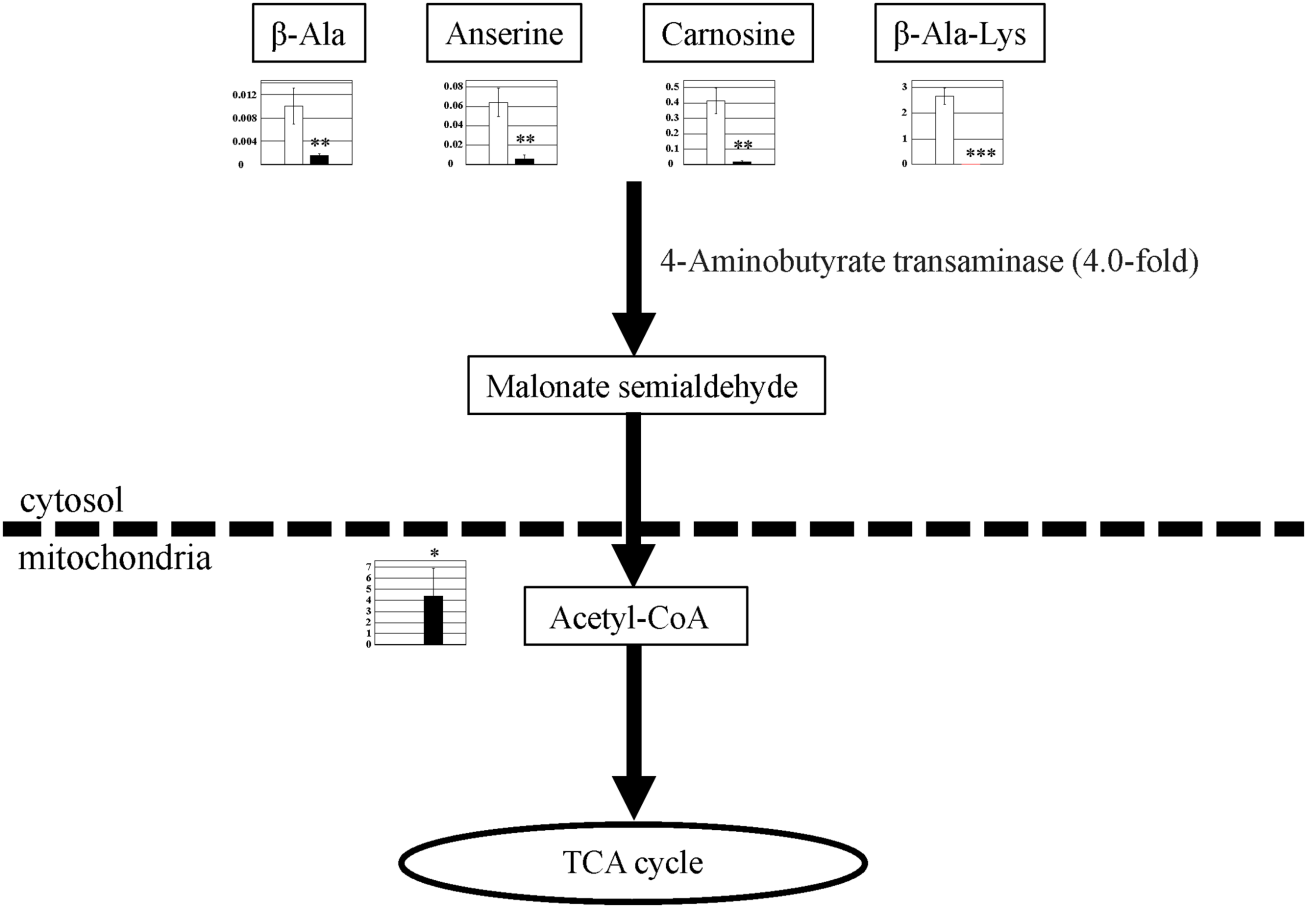
Observed metabolite changes mapped onto the pathways associated with β-alanine metabolism. Changes in the metabolite levels in the skeletal muscle of PGC-1α-Tg mice and WT mice are shown. Relative metabolite changes shown in the graphs were obtained by CE-TOFMS (S1 Table). Open bars, WT and filled bars, PGC-1α-Tg (N = 3). Data are expressed as the mean ± SD. Asterisks indicate statistically significant differences (***p < 0.001, **p < 0.01, *p < 0.05). Microarray data of gene expression change of enzymes and transporter in the related metabolic process are shown in the scheme.

Metabolic products related to β-alanine metabolism decreased in PGC-1α-Tg mice (Fig 9). This includes the levels of β-alanine (0.15-fold), anserine (dipeptide of β-alanine and methylhistidine) (0.08-fold), and carnosine (dipeptide of β-alanine and histidine) (0.04-fold). β-Ala–Lys (dipeptide of β-alanine and lysine) was detected in WT mice but not in PGC-1α-Tg mice (Fig 9 and S1 Table). β-alanine is metabolized by 4-aminobutyrate transaminase (4.0-fold in microarray) to malonate semialdehyde. Furthermore, acetyl-CoA (detected only in PGC-1α-Tg mice, but not in WT mice) is produced from malonate-semialdehyde by malonate-semialdehyde dehydrogenase (the probe for this enzyme was not present in the microarray). Thus, β-alanine is likely to be converted into acetyl-CoA and enter the TCA cycle.

### Other metabolite changes previously noted in the literature: neurotransmitters

The level of gamma-aminobutyric acid (GABA), a neurotransmitter, was reported to be increased in myocytes overexpressing PGC-1α [14]. In this study, it was also observed that there was an increased level of GABA (12-fold) in PGC-1α-Tg mice (S1 Table). PGC-1α-Tg is known to activate neural muscular junction function, including increased acetylcholine receptor gene expression [18]. In our microarray, we also observed increased acetylcholine receptor gene expression (Chnra1, 2.8-fold). Consistently, we observed increased acetylcholine levels in PGC-1α-Tg mice (detected only in PGC-1α-Tg mice, and not in WT mice, S1 Table). In addition, although the significance in skeletal muscle is not clear, we observed that another neurotransmitter, serotonin, was increased in PGC-1α-Tg mice (2.4-fold, S1 Table). The functional significance of increased neurotransmitters in PGC-1α-Tg mice needs to be investigated.

### Other metabolite changes previously noted in the literature: creatine

Brown et al. reported that PGC-1α up-regulates creatine transporter expression and creatine uptake in myotubes [19]. We observed increased creatine (1.2-fold), and decreased phosphocreatine (0.2-fold) and creatinine (0.6-fold) levels (S1 Table). Although creatine transporter gene expression did not change in our microarray, expression of creatine kinase (mitochondrial 2, Ckmt2) (creatine phosphate + ADP ←→ creatine + ATP) increased in PGC-1α-Tg mice (4.4-fold). Creatine plays an important role in skeletal muscle energy production during exercise [19, 20]. PGC-1α may be involved in the metabolism of creatine/phosphocreatine.

### Possible metabolic effect of long-term-exercise-induced PGC1α

We have to be careful that the expression level of PGC1α driven by the PGC1α transgene in PGC1α-Tg mice remains high after birth. The results of the metabolomic analysis conducted in this study may not be applicable to the metabolic changes by physiological increases in PGC1α level observed in other contexts, such as exercise in wild-type mice. On the other hand, Egan et al. reported that exercise-induced increases in mRNA levels are a temporary response and are not translated into protein during the bout of exercise. Superimposition of repeated exercise bouts results in the general accumulation of protein in response to repeated, pulsed increases in relative mRNA expression [21]. The PGC1α-Tg mice in this study may be an appropriate model of the effects of long-term exercise. This remains clarified in future study.

### PGC1α-b and other isoforms of PGC1α

We used PGC1α-b transgenic mice in this study, as described in the Methods section. PGC1α-b is an isoform of PGC1α. The PGC1α-b, which is considered to be similar to PGC1α-a (the originally found to be full-length PGC-1α) [1] in function, structurally differs by 16 amino acids at its amino terminus. Moreover, transcriptional activity did not differ among PGC1α-a, PGC1α-b, and PGC1α-c in a reporter assay [22]. Furthermore, gene expression changes were similar among lines of PGC1α-a and PGC1α-b mice (S2 Table). We consider that changes in the PGC1α-b Tg mice are representative of the effects of full-length PGC1α (not isoform-specific). However, we cannot exclude the possibly that there may be some PGC1α-b isoform-specific effects. This remains to be clarified in a future study.

### Conclusion

**Fig 10:**
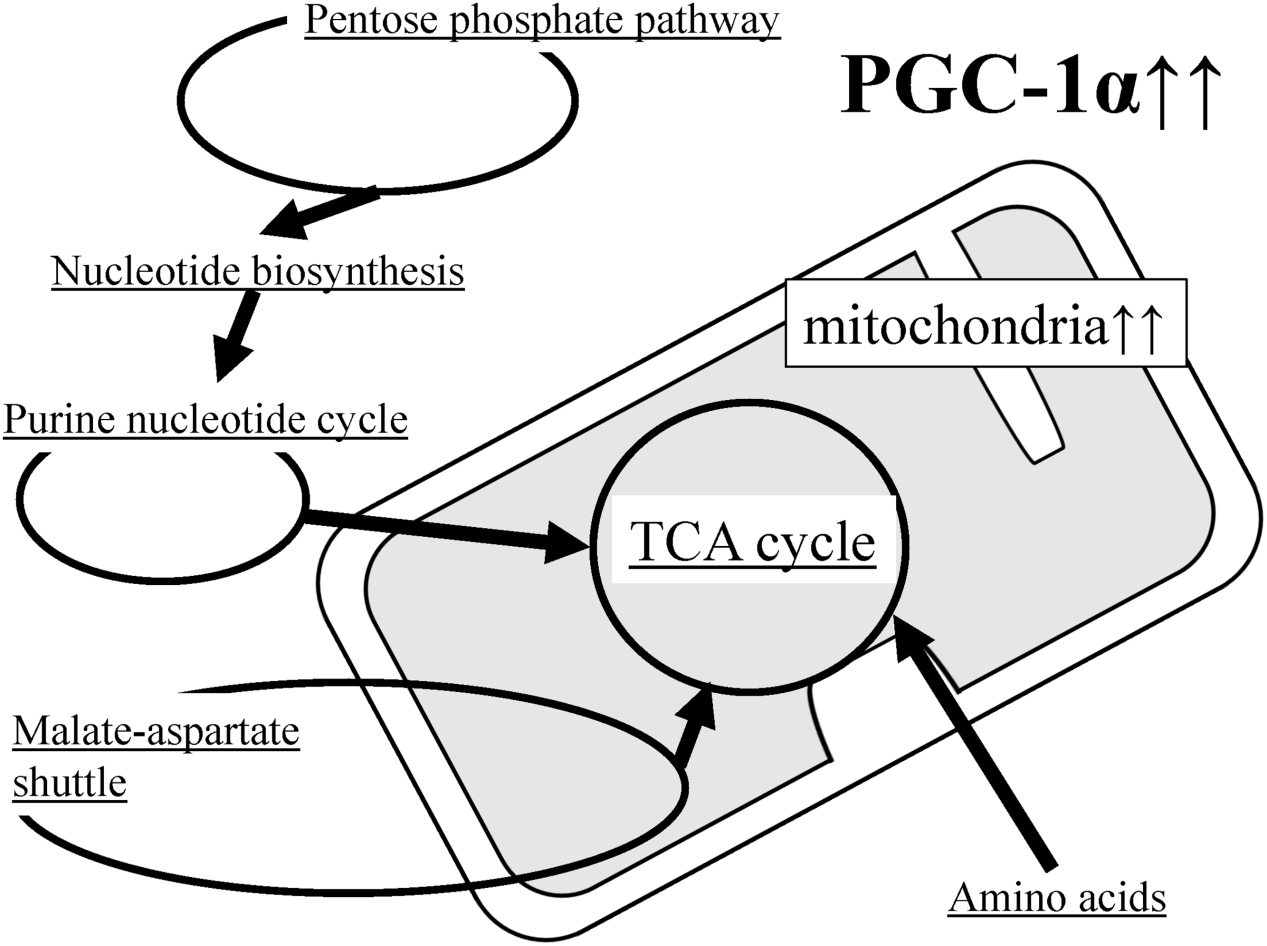
Schematic representation of metabolic pathway changes in PGC-1α-Tg mice. The levels of many metabolic products are changed in the skeletal muscle of PGC-1α-Tg mice. Many of these changes are associated with mitochondrial metabolism, in particular the TCA cycle. Increased mitochondrial content due to PGC-1α-overexpression appears to activate the TCA cycle (Fig 3); therefore, there must be more substrates available for the TCA cycle. For instance, the activated pentose phosphate pathway (Fig 4) stimulates nucleotide synthesis (Fig 5), which is followed by activation of the purine nucleotide cycle (Fig 6), supplying fumarate for the TCA cycle. Meanwhile, activation of the malate-aspartate shuttle supplies other substrates (Fig 7). In addition, amino acids are also likely to be used as substrates (Figs 7, 8 and 9). Increased coordinated regulation of the TCA cycle and amino acid metabolism, including BCAA, suggests that PGC-1α plays important roles in energy metabolism. Moreover, activation of the purine nucleotide pathway and malate–aspartate shuttle, which are known to be active during exercise, further suggests that PGC-1α regulates metabolism in exercise.

In this study, it was observed that many metabolic product levels changed in the skeletal muscle of PGC-1α-Tg mice (Fig 10). Many of these changes are related to mitochondrial metabolism. Increased coordinal regulation of the TCA cycle and amino acid metabolism, including BCAA, suggests that PGC-1α plays important roles in energy metabolism. Moreover, activation of the purine nucleotide pathway, malate–aspartate shuttle as well as creatine metabolism, which are known to be active during exercise, further suggests that PGC-1α regulates metabolism in exercise. In this study, we evaluated the role of PGC-1α in the skeletal muscle at the metabolic level.

## Methods

### Transgenic (Tg) mice

Tg mice overexpressing PGC-1α in the skeletal muscle (PGC-1α-Tg mice) were generated as described [22]. In brief, the human α-skeletal actin promoter was used to express PGC-1α in the skeletal muscle (C57BL/6 background). We used PGC1α-b (B) mice [4], which is same to PGC1α-b (03–2) mice [22]. Two independent lines of Tg mice (PGC1α-b (B)/ PGC1α-b (03–2) mice and PGC1α-b (A)/ PGC1α-b (02–1) mice) [22] showed similar phenotypes in a previous study [4]. The mice were maintained in a controlled environment with a constant temperature of 24°C, with fixed artificial light (12-h light–dark cycle).

### Metabolomic analysis

Gastrocnemius muscles of male PGC-1α-Tg mice and sex-matched WT control mice littermates were used for metabolomic analysis (Human Metabolome Technologies Inc., Tsuruoka, Japan) [23, 24]. The age of mice used were: WT1, 13 weeks; WT2, 10 weeks; WT3, 9 weeks; Tg1, 13 weeks; Tg2, 10 weeks; and Tg3, 9 weeks of age. Three mice each were used in the PGC-1α-Tg and WT groups. Frozen mice muscle samples were transferred into 500 μl of methanol containing 50 mM of the external standard. After homogenization by BMSM10N21 (BMS, Tokyo) at 1,500 rpm for 120 s performed five times, 500 μl of chloroform and 200 μl of ultrapure water were added to the homogenate and mixed well and centrifuged at 2,300 g for 5 min at 4°C. The resultant water phase was ultrafiltrated by the Millipore Ultrafree-MC PLHCC HMT Centrifugal Filter Device, 5 kDa (Millipore, Billerica, MA). The filtrates were then dried and dissolved in 50 μl of ultrapure water. The samples obtained were then subjected to capillary electrophoresis time-of-flight mass spectrometry (CE-TOFMS) analysis using the Agilent CE-TOFMS system (Agilent Technologies, Santa Clara, CA) at 4°C. The detected peaks were aligned according to their m/z values and normalized migration times. The peaks were mean-centered and scaled using their standard deviations on a per-peak basis as a pretreatment. After applying autoscaling, a principal component analysis (PCA) and a hierarchical clustering analysis (HCA) were conducted using JMP ver. 11 software (SAS Institute, Cary, North Carolina, USA). In the PCA, a score plot of the first and second principal components was generated. In the HCA, the resulting data sets from each genotype were clustered by Euclidean distance using Ward’s method [25]. Heat maps were generated by coloring the values of all data across their value ranges. The relative area of each peak was calculated and used for the comparison between the PGC1α-Tg and WT groups.

### Ethics Statement

Mice were cared for in accordance with the National Institutes of Health (NIH) Guide for the Care and Use of Laboratory Animals and our institutional guidelines. All animal experiments were performed with the approval of the Institutional Animal Care and Use Committees of the University of Shizuoka and Kyoto Prefectural University. All surgery was performed under sodium pentobarbital anesthesia, and all efforts were made to minimize suffering.

### cDNA microarray analysis

cDNA microarray data were collected as described previously [8]. Briefly, RNA was isolated from skeletal muscle (gastrocnemius) of PGC-1α-Tg mice and age- and sex-matched WT control mice. Samples from WT and PGC-1α-Tg mice (N = 5) were pooled and used. Each sample was labeled with a cyanine 3-CTP using the Low Input Quick Amp Labeling Kit (Agilent Technologies, Inc., Santa Clara, CA) and hybridized to the Agilent whole mouse genome microarray (4× 44K), which contains 41,534 genes including expressed sequence tags. Signal detection and data analysis were performed according to the manufacturer’s instructions. The microarray data was submitted to GEO database (accession No. GSE67049).

### Quantitative real-time RT-PCR analysis

Total RNA was prepared usnig TRIzol (Life Technologies Japan, Tokyo, Japan). cDNA was synthesized from 1 μg of total RNA using the ReverTra Ace qPCR RT Master Mix. Transcription Kit (TOYOBO, Tokyo, Japan). Gene expression levels were measured as described [8]. The mouse-specific primer pairs used were as shown in Table 1. Results of real-time RT-PCR analysis were shown in S1 Fig.

**Table 1:** Mouse-specific primer pairs used for quantitative real-time RT-PCR.

Gene	Forward	Reverse
PGC1a	CGGAAATCATATCCAACCAG	TGAGGACCGCTAGCAAGTTTG
Citrate synthase	AAGTTGGCAAAGACGTGTCAG	TACTGCATGACCGTATCCTGG
Aconitase	TGGCTGCCAGTATGACCAAGT	ATGTGGCTTTAGCTCATTGAGGTT
Isocitrate dehydrogenase	ATTTTGTGGTAGATCGAGCTGG	CCTCCGGCAGGGAAGTTATAC
Succinate dehydrogenase	CCTCGAATGCAGACGTACGA	CAACACCATAGGTCCGCACTT
Malate dehydrogenase 2	AAGGCTACCTTGGACCGGAG	CATCACAACCTTTGAGGCAATCT
Glucose-6-phosphate dehydrogenase	ATCATCATGGGTGCATCGG	GGTAGGATAGATCTTCTTCTTGGCC
Adenylosuccinate lyase	TCTGCCCACGTTAGGTTTCAC	CGCTTCAAGTTCTGGAGATCC
AMP deaminase 3	GTGGAGATTACTGTGCAGGGATC	TGGCAGCCTGCTCATAGTCTT
Adenylate kinase 3	TCACGAGCTCAAAACCCTTACC	GGAAATCCATCCAACAGCCA
Glutamate oxalate transaminase 1	CCAACCTGGGAGAACCATAATG	CCAGTAGCAATAGGGCCGAAT
Malate dehydrogenase 1	TGTTGTACAGTATTGGAAATGGATCTG	TGATGGGCTGGTCTTTCCC
Slc25a13	ACTCTGGCTGGCAACAGGAA	CCAAAGCGAACTCCTCCTTAGTC
Slc25a11	ACACTGGGCTGTCAGCTGGT	CGAGTAGTGGTGTAGGTGGCCT
Branched chain aminotransferase 2	CGGACCCTTCATTCGTCAGA	CCATAGTTCCCCCCCAACTT
Glutamate oxalate transaminase 2	GATCCGTCCCCTGTATTCCA	CACCTCTTGCAACCATTGCTT
Threonine aldorase	GGAGGTGCTACCAAGGGACC	GAGTCCTTTAGCGAATCTCTGGG
Serine hydroxylmethyltransferase	AGTGATGCCGAGGTTTACAGC	CCGAGGCAATCAGCTCTAATC
Serine dehydratase	GTCTCCCCGTTTGACCATCC	GGGGTCCCTAGTGACTCCTTC
Alanine transaminase	GCGCCAGGGTGTGAAGAA	GCTTGTGCATCCCCAATATTG
Pyruvate dehydratase	TCAGCACTCGCAATGCTTTG	ATAAGTCCTTTTGCATCCTCGG
4-Aminobutyrate transaminase 2	TTGTTGATTACCCGACGGCT	GAGGTGGAGGTTTTTCTGGGA

## Supporting Information

S1 Fig Gene expression of the gene changed in skeletal muscle of PGC-1α-Tg mice. Gene expression of A) PGC-1α and pathway in B) TCA cycle (Fig 3), C) pentose phosphate pathway (Fig 4), D) purine nucleotide cycle (Fig 6), E) BCAA metabolism and malate-aspartate shuttle (Fig 7), F) the pathway associated with glycine, threonine, serine and alanine metabolism (Fig 8), G) β-alanine metabolism (Fig 9) genes in WT (control; open columns, N = 4) and PGC-1α-Tg (filled columns, N = 6) mice by quantitative real-time RT-PCR. *** P < 0.001, ** P < 0.01, * P < 0.05.
(PDF)

S1 Table List of metabolites detected in CE-TOFMS. “Relative area” is the peak value of each metabolite normalized by sample volume; i.e., relative concentration of each metabolite. “Mean” is the mean value of the relative area from each group [WT and PGC-1α-Tg (N = 3)]. “Ratio” is the comparative value of the relative areas (PGC-1α-Tg per WT). “Not detected in WT” or “Not detected in Tg” means the peak of the metabolite was below the detection level in WT or Tg samples. P-value is calculated by Studentʼs T-test (***p < 0.001, **p < 0.01, *p < 0.05).
(PDF)

S2 Table List of gene expression change of transgenic lines of PGC1α-b (B), PGC1α-b (A)/ PGC1α-b (02–1) and PGC1α-b (B)/ PGC1α-b (03–2) in microarray. Fold changes of relative gene expression in skeletal muscle of each transgenic line compared with wild-type control mice are shown. Listed are genes used in the Figs 3–9. The data of PGC1α-b (B)/ PGC1α-b (03–2) are shown in the Figs 3–9.
(PDF)
