# Supplementary material for: Metabolomic Analysis of the Skeletal Muscle of Mice Overexpressing PGC-1α
Source: PLoS One. 2015 Jun 26;10(6):e0129084. doi: 10.1371/journal.pone.0129084 (PMC4482640; doi:10.1371/journal.pone.0129084)
Supplement: S1 Fig — Gene expression of A) PGC-1α and pathway in B) TCA cycle (Fig 3), C) pentose phosphate pathway (Fig 4), D) purine nucleotide cycle (Fig 6), E) BCAA metabolism and malate-aspartate shuttle (Fig 7), F) the pathway associated with glycine, threonine, serine and alanine metabolism (Fig 8), G) β-alanine metabolism (Fig 9) genes in WT (control; open columns, N = 4) and PGC-1α-Tg (filled columns, N = 6) mice by quantitative real-time RT-PCR. *** P < 0.001, ** P < 0.01, * P < 0.05. (PDF) [file pone.0129084.s001.pdf]

A

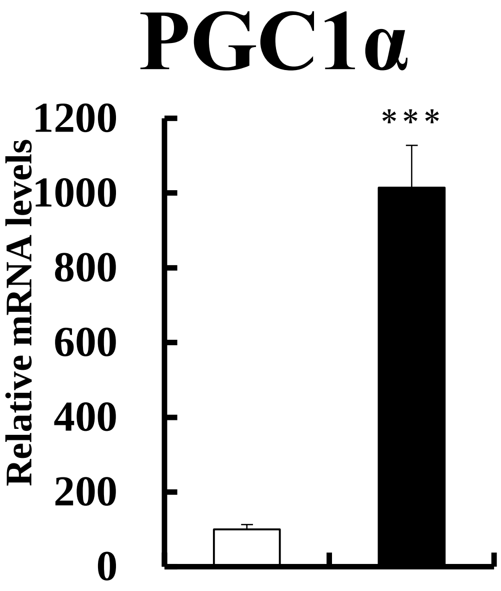

B

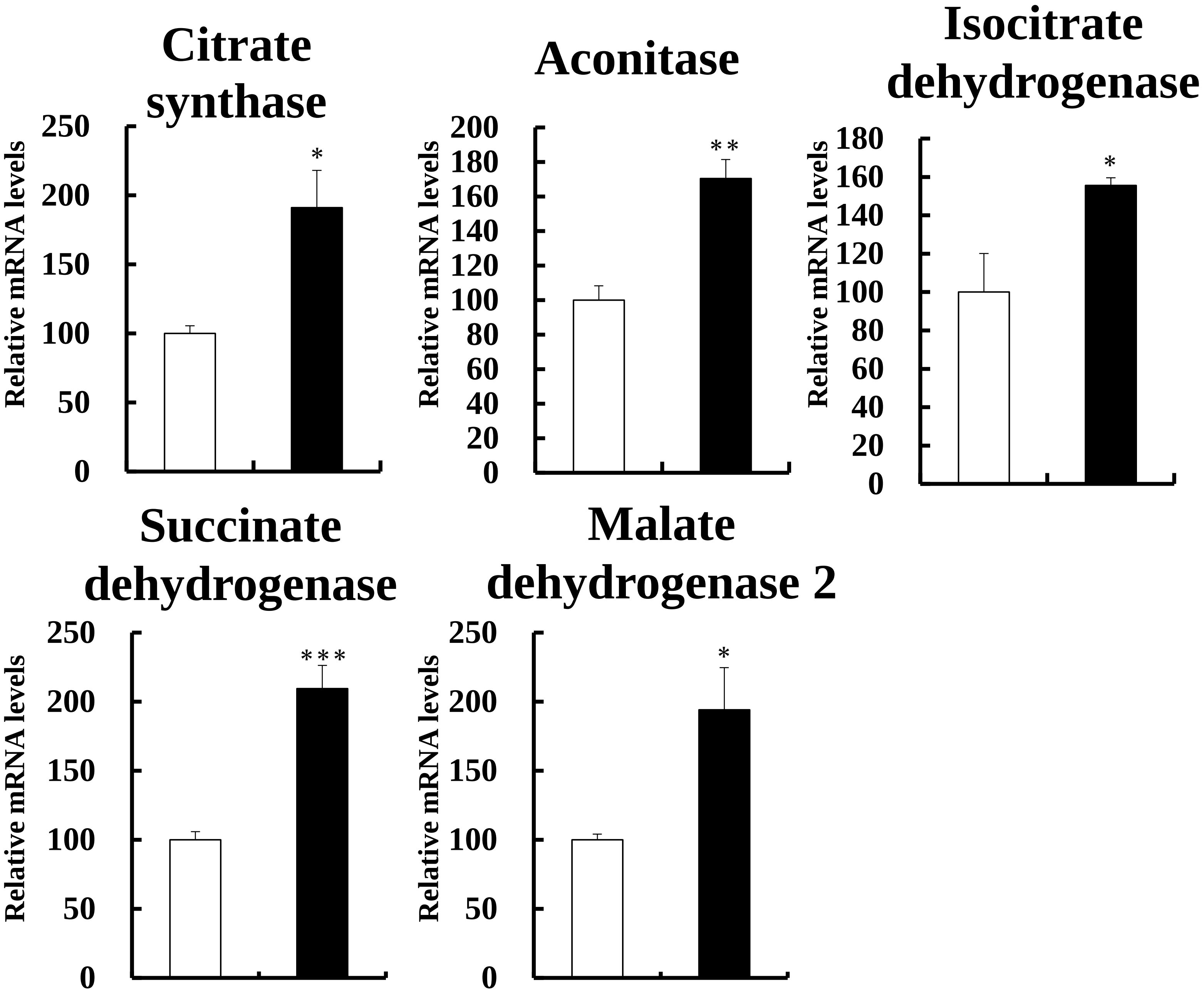

Citrate synthase
Aconitase
Isocitrate dehydrogenase
Succinate dehydrogenase
Malate dehydrogenase 2
Relative mRNA levels
0
50
100
150
200
250
20
40
60
80
120
140
160
180
*
**
***

C

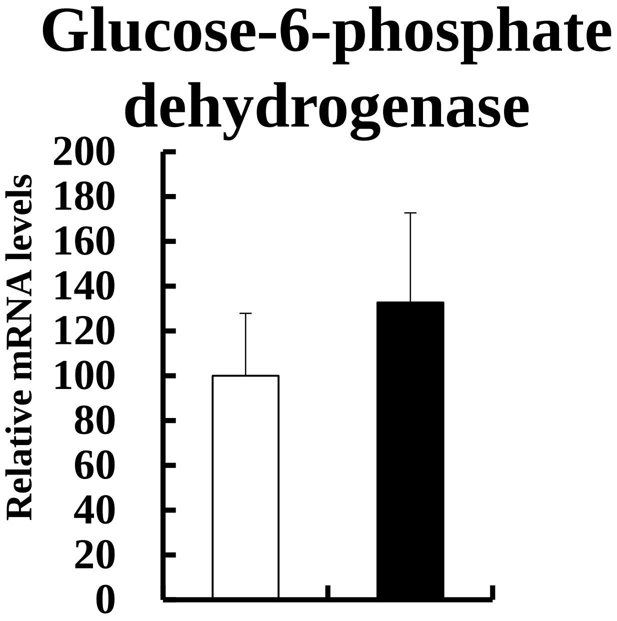
Glucose-6-phosphate
dehydrogenase
Relative mRNA levels
200
180
160
140
120
100
80
60
40
20
0

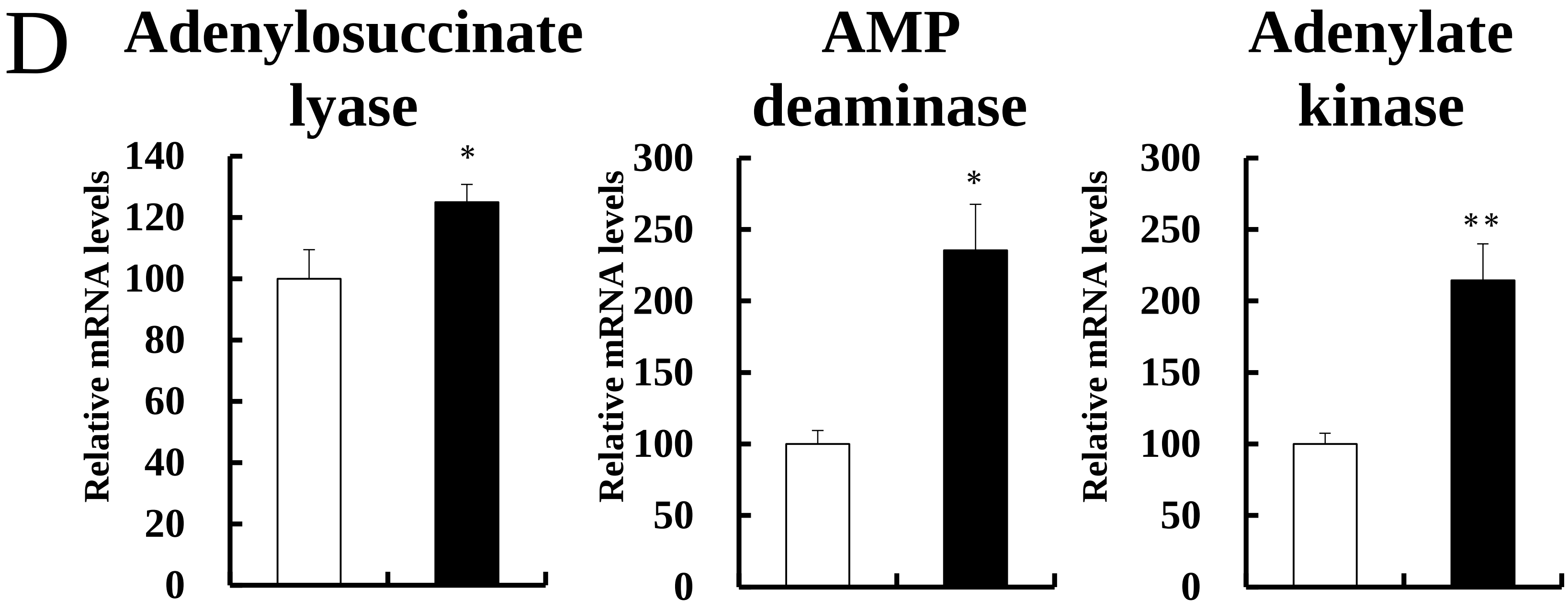
D
Adenylosuccinate lyase
AMP deaminase
Adenylate kinase
Relative mRNA levels
140
120
100
80
60
40
20
0
300
250
200
150
100
50
*
**

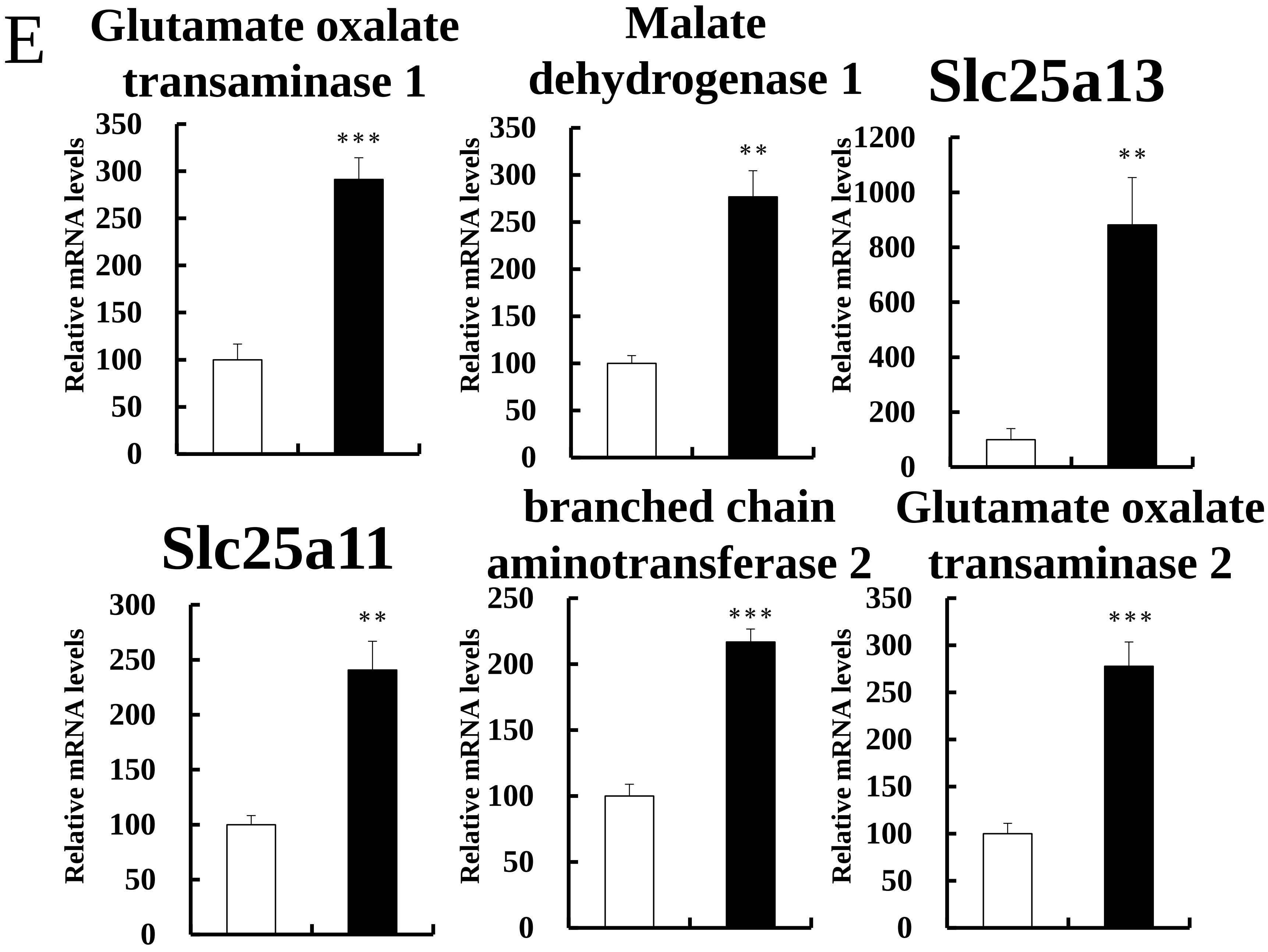
E
Glutamate oxalate transaminase 1
Malate dehydrogenase 1
Slc25a13
Slc25a11
branched chain aminotransferase 2
Glutamate oxalate transaminase 2
Relative mRNA levels
***
**
0
50
100
150
200
250
300
350
400
600
800
1000
1200

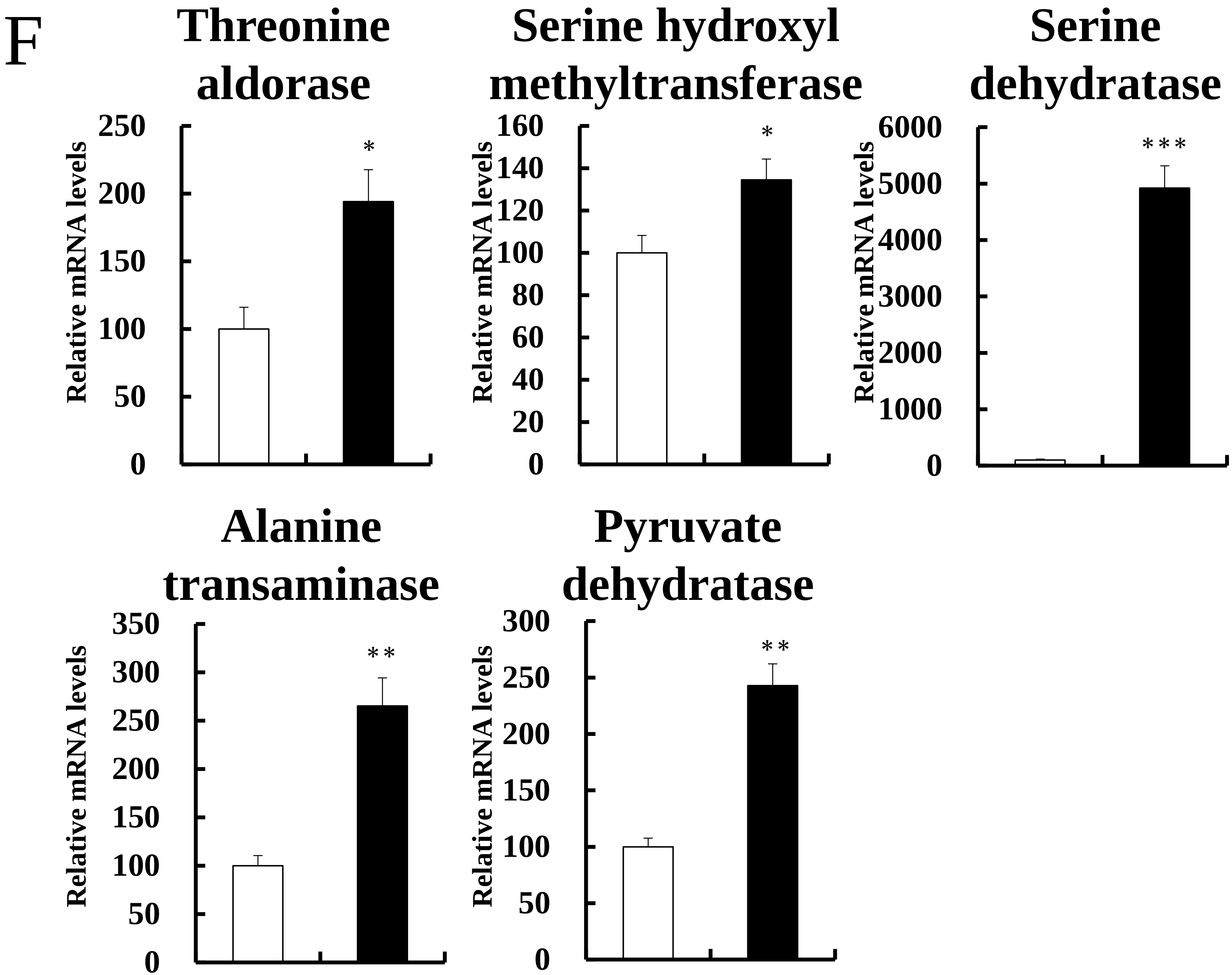

F
Threonine aldorase
Serine hydroxyl methyltransferase
Serine dehydratase
Alanine transaminase
Pyruvate dehydratase
Relative mRNA levels
*
***
**

G

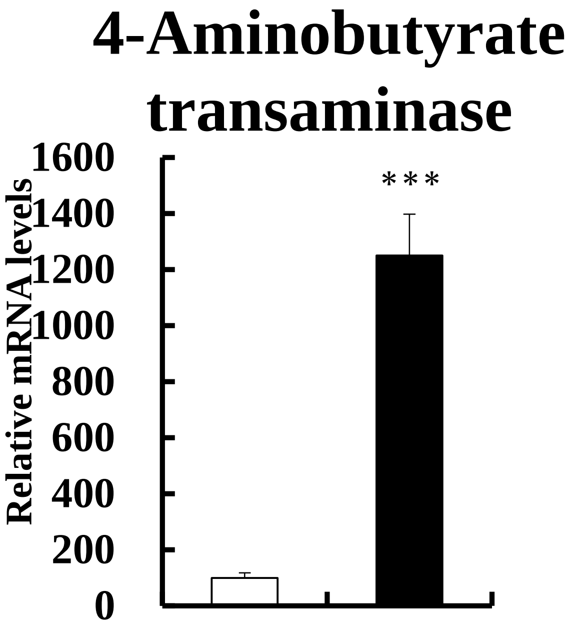

S1 Fig. Gene expression of the gene changed in skeletal muscle of PGC-1 α -Tg mice
Gene expression of A) PGC-1 α and pathway in B) TCA cycle (Fig.3), C) pentose phosphate pathway (Fig.4), D) purine nucleotide cycle (Fig.6), E) BCAA metabolism and malate-aspartate shuttle (Fig.7), F) the pathway associated with glycine, threonine, serine and alanine metabolism (Fig.8), G) β -alanine metabolism (Fig.9) genes in WT (control; open columns, N = 4) and PGC-1 α -Tg (filled columns, N = 6) mice by quantitative real-time RT-PCR. *** $P < 0.001$, ** $P < 0.01$, * $P < 0.05$.
