# Supplementary material for: Metabolomic Analysis of the Skeletal Muscle of Mice Overexpressing PGC-1α
Source: PLoS One. 2015 Jun 26;10(6):e0129084. doi: 10.1371/journal.pone.0129084 (PMC4482640; doi:10.1371/journal.pone.0129084)
Supplement: S1 Table — “Relative area” is the peak value of each metabolite normalized by sample volume; i.e., relative concentration of each metabolite. “Mean” is the mean value of the relative area from each group [WT and PGC-1α-Tg (N = 3)]. “Ratio” is the comparative value of the relative areas (PGC-1α-Tg per WT). “Not detected in WT” or “Not detected in Tg” means the peak of the metabolite was below the detection level in WT or Tg samples. P-value is calculated by Studentʼs T-test (***p < 0.001, **p < 0.01, *p < 0.05). (PDF) [file pone.0129084.s002.pdf]

Compound name	Relative Area								Comparative Analysis		
	WT			PGC-1α-Tg			Mean		PGC-1α-Tg vs WT		
	WT1	WT2	WT3	Tg1	Tg2	Tg3	WT	PGC-1α-Tg	Ratio	*p* -value	
Hexanoic acid	0.000	0.000	0.000	0.207	0.467	0.000	0.000	0.337	not detected in WT	0.172	
Mevalonic acid	0.000	0.000	0.000	0.257	0.320	0.738	0.000	0.438	not detected in WT	0.044	*
p-Hydroxymandelic acid	0.000	0.000	0.000	0.157	0.259	0.399	0.000	0.271	not detected in WT	0.018	*
Glyceraldehyde 3-phosphate	0.000	0.000	0.000	0.000	0.488	0.000	0.000	0.488	not detected in WT	0.374	
Ascorbic acid	0.000	0.000	0.000	0.000	0.316	1.424	0.000	0.870	not detected in WT	0.250	
Sebacic acid	0.000	0.000	0.000	0.000	0.000	0.317	0.000	0.317	not detected in WT	0.374	
CMP	0.000	0.000	0.000	0.000	0.918	0.647	0.000	0.782	not detected in WT	0.128	
UMP	0.000	0.000	0.000	0.000	2.795	2.691	0.000	2.743	not detected in WT	0.116	
AMP	0.000	0.000	0.000	0.213	28.514	2.256	0.000	10.327	not detected in WT	0.320	
UDP	0.000	0.000	0.000	0.241	1.635	0.722	0.000	0.866	not detected in WT	0.102	
Acetyl CoA divalent	0.000	0.000	0.000	0.179	0.459	0.677	0.000	0.438	not detected in WT	0.038	*
GDP	0.000	0.000	0.000	0.000	2.254	0.682	0.000	1.468	not detected in WT	0.216	
Trimethylamine	0.000	0.000	0.000	0.000	0.000	1.407	0.000	1.407	not detected in WT	0.374	
3-Aminoisobutyric acid	0.000	0.000	0.000	0.746	0.685	0.955	0.000	0.795	not detected in WT	0.001	***
Uracil	0.000	0.000	0.000	0.000	0.000	0.351	0.000	0.351	not detected in WT	0.374	
Ectoine	0.000	0.000	0.000	0.000	0.000	0.188	0.000	0.188	not detected in WT	0.374	
Acetylcholine	0.000	0.000	0.000	0.094	0.154	0.116	0.000	0.121	not detected in WT	0.002	**
threo-β-Methylaspartic acid	0.000	0.000	0.000	0.154	0.178	0.167	0.000	0.166	not detected in WT	0.000	***
Ala-Ala	0.000	0.000	0.000	0.285	0.563	0.469	0.000	0.439	not detected in WT	0.006	**
N-Acetylornithine	0.000	0.000	0.000	0.000	0.205	0.160	0.000	0.183	not detected in WT	0.122	
N8-Acetylspermidine	0.000	0.000	0.000	0.000	0.094	0.107	0.000	0.101	not detected in WT	0.118	
γ-Glu-Cys	0.000	0.000	0.000	0.000	0.137	0.229	0.000	0.183	not detected in WT	0.140	
GMP	0.000	0.074	0.059	0.094	1.314	1.864	0.066	1.090	16.46	0.116	
GABA	0.223	0.415	0.384	3.298	4.384	4.717	0.340	4.133	12.14	0.001	***
Adenylosuccinic acid	0.081	0.177	0.166	0.394	1.817	2.860	0.141	1.690	11.95	0.096	
CoA divalent	0.197	0.166	0.000	1.151	1.235	2.114	0.182	1.500	8.25	0.012	*
Adenosine	0.254	0.338	0.497	2.284	1.946	1.768	0.363	1.999	5.51	0.001	***
IMP	4.846	31.058	8.919	29.167	74.115	140.274	14.941	81.185	5.43	0.117	
FAD divalent	0.221	0.278	0.196	1.019	1.206	1.209	0.232	1.144	4.94	0.000	***
Thiamine diphosphate	0.122	0.158	0.182	0.692	0.839	0.720	0.154	0.750	4.88	0.000	***
NADH	0.175	0.110	0.217	0.592	0.962	0.799	0.168	0.784	4.68	0.005	**
Pantothenic acid	0.724	0.694	0.761	2.170	3.656	4.253	0.726	3.360	4.63	0.013	*
3',5'-ADP	0.000	0.000	0.198	0.535	0.951	0.911	0.198	0.799	4.03	0.008	**
Isethionic acid	0.609	0.622	0.605	2.359	2.824	2.217	0.612	2.467	4.03	0.001	***
Ethanolamine phosphate	1.528	1.600	1.632	3.646	8.019	6.690	1.587	6.118	3.86	0.025	*
myo-Inositol 1-phosphate<br>myo-Inositol 3-phosphate	2.439	2.904	2.566	8.658	11.876	9.154	2.636	9.896	3.75	0.002	**
Glycerophosphocholine	4.447	5.240	4.470	11.744	19.619	20.147	4.719	17.170	3.64	0.010	*
ADP-ribose	1.655	1.391	1.350	4.146	5.972	5.719	1.465	5.279	3.60	0.003	**
Ribulose 5-phosphate	0.799	0.963	0.724	1.973	2.790	4.125	0.829	2.962	3.58	0.028	*
Hippuric acid	0.000	0.000	0.052	0.000	0.206	0.166	0.052	0.186	3.57	0.178	
ADP	3.846	17.489	4.247	8.555	62.056	17.490	8.527	29.367	3.44	0.291	
Hypotaurine	4.826	5.181	6.110	15.808	14.482	21.441	5.373	17.244	3.21	0.005	**
Glu	77.439	84.045	91.802	209.112	303.557	263.841	84.428	258.837	3.07	0.003	**
NADP+	0.482	0.403	0.509	1.270	1.504	1.470	0.465	1.415	3.05	0.000	***
Argininosuccinic acid	0.178	0.142	0.146	0.333	0.458	0.620	0.156	0.470	3.02	0.020	*
Inosine	1.508	2.357	1.348	3.561	4.676	7.248	1.738	5.162	2.97	0.039	*
N-Acetylaspartic acid	0.455	0.616	0.531	1.243	2.073	1.367	0.534	1.561	2.92	0.017	*
N-Acetylglucosamine 1-phosphate	0.000	0.000	0.131	0.382	0.424	0.340	0.131	0.382	2.91	0.003	**
N-Acetyllysine	0.000	0.256	0.000	0.461	0.912	0.794	0.256	0.722	2.82	0.016	*
Fumaric acid	3.089	3.160	3.838	7.984	9.679	10.587	3.362	9.417	2.80	0.002	**
Nicotinamide	19.919	18.039	14.287	42.191	48.630	53.565	17.415	48.129	2.76	0.001	**
2-Aminoadipic acid	0.508	0.727	0.508	1.311	1.616	1.663	0.581	1.530	2.63	0.002	**
Glycerol 3-phosphate	21.380	23.471	33.823	64.811	63.679	72.782	26.225	67.091	2.56	0.001	**
CDP-choline	0.060	0.082	0.100	0.181	0.203	0.229	0.081	0.204	2.53	0.002	**
Histamine	2.910	2.116	1.622	6.071	6.465	3.995	2.216	5.510	2.49	0.018	*
Guanidoacetic acid	0.440	0.501	0.434	0.841	1.356	1.189	0.458	1.129	2.46	0.012	*
UDP-glucuronic acid	0.231	0.276	0.181	0.269	0.688	0.714	0.229	0.557	2.43	0.089	
N-Acetylneuraminic acid	0.125	0.168	0.176	0.268	0.411	0.454	0.156	0.378	2.42	0.019	*
Ribose 5-phosphate	0.231	0.302	0.185	0.386	0.635	0.695	0.239	0.572	2.39	0.030	*
Serotonin	0.045	0.000	0.031	0.078	0.106	0.087	0.038	0.090	2.39	0.014	*
Malonylcarnitine	0.082	0.092	0.105	0.179	0.233	0.255	0.093	0.222	2.38	0.005	**
Gluconic acid	0.288	0.276	0.388	0.000	0.729	0.762	0.317	0.746	2.35	0.514	
Ergothioneine	0.000	0.000	1.570	1.639	5.899	3.386	1.570	3.641	2.32	0.081	
Thiamine	0.033	0.000	0.025	0.059	0.081	0.062	0.029	0.067	2.32	0.017	*
Citric acid	15.609	16.771	12.113	33.013	36.380	33.578	14.831	34.324	2.31	0.000	***
Glutathione (GSH)	31.015	13.629	38.165	35.044	57.074	98.395	27.603	63.504	2.30	0.146	
UDP-glucose<br>UDP-galactose	0.687	1.228	1.073	1.669	2.719	2.480	0.996	2.289	2.30	0.022	*
Malic acid	23.485	22.361	24.625	52.186	49.738	58.542	23.491	53.489	2.28	0.000	***
Homovanillic acid	0.000	0.130	0.000	0.303	0.294	0.275	0.130	0.291	2.23	0.005	**
Asp	21.459	15.274	20.492	35.747	48.141	42.820	19.075	42.236	2.21	0.005	**
UDP-N-acetylgalactosamine<br>UDP-N-acetylglucosamine	1.145	1.441	1.511	2.265	3.435	3.277	1.366	2.992	2.19	0.013	*
Uric acid	0.490	0.421	0.344	0.696	1.105	0.930	0.418	0.910	2.18	0.017	*
Choline	21.935	15.308	11.565	37.108	35.915	32.145	16.269	35.056	2.15	0.005	**
Succinic acid	19.880	22.505	27.678	41.285	53.743	55.333	23.354	50.120	2.15	0.006	**
3-Indoxylsulfuric acid	0.062	0.079	0.000	0.114	0.183	0.154	0.070	0.150	2.14	0.030	*
Gly-Gly	0.232	0.273	0.264	0.512	0.589	0.474	0.256	0.525	2.05	0.002	**
Fructose 1,6-diphosphate	31.631	15.230	43.283	43.545	69.031	70.639	30.048	61.072	2.03	0.061	
Ethanolamine	2.230	2.044	1.340	3.823	4.210	3.370	1.871	3.801	2.03	0.006	**
Homoserinelactone	0.103	0.131	0.099	0.203	0.242	0.231	0.111	0.225	2.02	0.002	**
NADPH divalent	0.122	0.125	0.153	0.343	0.311	0.144	0.133	0.266	2.00	0.101	
Spermine	0.204	0.258	0.330	0.382	0.529	0.637	0.264	0.516	1.96	0.037	*
2-Hydroxyglutaric acid	1.440	1.213	0.434	2.652	2.386	0.922	1.029	1.987	1.93	0.196	
S-Adenosylmethionine	0.659	0.783	0.760	1.152	1.481	1.423	0.734	1.352	1.84	0.005	**
trans-Glutaconic acid	0.494	0.612	0.000	1.036	0.988	0.000	0.553	1.012	1.83	0.473	
N6-Methyllysine	1.234	1.806	2.166	2.393	3.404	3.727	1.736	3.175	1.83	0.041	*
CMP-N-acetylneuraminate	0.402	0.486	0.563	0.794	0.858	0.929	0.484	0.860	1.78	0.003	**
Carnitine	55.622	56.796	82.508	100.297	126.137	119.211	64.976	115.215	1.77	0.013	*
Nω-Methylarginine	0.000	0.112	0.119	0.139	0.197	0.270	0.115	0.202	1.75	0.081	
Dihydroxyacetone phosphate	21.399	11.363	27.195	20.852	43.066	40.863	19.986	34.927	1.75	0.152	
Putrescine	0.194	0.172	0.376	0.217	0.473	0.607	0.248	0.432	1.75	0.232	
Carboxymethyllysine	0.302	0.360	0.347	0.473	0.647	0.641	0.336	0.587	1.75	0.013	*
Arg	30.748	37.822	37.183	52.926	50.707	77.949	35.251	60.527	1.72	0.049	*
Gln	198.874	188.818	204.712	295.731	365.361	331.464	197.468	330.852	1.68	0.003	**
O-Acetylcarnitine	37.547	42.603	47.033	66.901	74.148	71.727	42.394	70.925	1.67	0.001	**
Glu-Glu	0.054	0.056	0.052	0.079	0.104	0.086	0.054	0.090	1.66	0.009	**
Lys	46.761	69.996	64.712	81.879	89.892	126.915	60.490	99.562	1.65	0.066	
SDMA	0.088	0.131	0.085	0.150	0.163	0.176	0.101	0.163	1.62	0.020	*

5-Hydroxylysine	0.264	0.279	0.391	0.404	0.388	0.713	0.311	0.502	1.61	0.168	
N-Glycolylneuraminic acid	0.139	0.183	0.259	0.215	0.317	0.379	0.194	0.303	1.57	0.138	
Trimethylamine N-oxide	0.880	0.992	1.046	1.027	1.939	1.458	0.973	1.475	1.52	0.134	
GDP-glucose	0.160	0.231	0.173	0.223	0.309	0.314	0.188	0.282	1.50	0.064	
Spermidine	0.854	0.863	1.662	0.952	1.750	2.128	1.126	1.610	1.43	0.331	
6-Phosphogluconic acid	0.247	0.251	0.225	0.194	0.274	0.556	0.241	0.341	1.41	0.416	
Adenine	0.483	0.496	0.469	0.679	0.690	0.655	0.483	0.675	1.40	0.000	***
Cytidine	0.257	0.253	0.171	0.273	0.394	0.272	0.227	0.313	1.38	0.156	
Diethanolamine	0.316	0.322	0.349	0.601	0.496	0.263	0.329	0.453	1.38	0.284	
γ-Butyrobetaine	3.787	4.760	5.319	6.400	6.075	6.145	4.622	6.206	1.34	0.026	*
Butyrylcarnitine	0.924	1.065	0.751	1.212	1.070	1.364	0.914	1.215	1.33	0.072	
4-Guanidinobutyric acid	0.159	0.206	0.260	0.177	0.306	0.342	0.208	0.275	1.32	0.314	
Sedoheptulose 7-phosphate	0.106	0.000	0.132	0.130	0.181	0.151	0.119	0.154	1.29	0.159	
GTP	4.769	5.480	5.144	6.808	6.116	6.727	5.131	6.550	1.28	0.009	**
NAD+	17.211	20.722	25.323	26.384	27.920	26.037	21.085	26.780	1.27	0.078	
Glutathione (GSSG) divalent	46.317	54.281	44.533	69.224	63.125	51.150	48.377	61.166	1.26	0.104	
1-Methylnicotinamide	0.158	0.135	0.276	0.182	0.311	0.227	0.190	0.240	1.26	0.434	
Trigonelline	0.673	0.653	0.738	0.819	0.761	0.999	0.688	0.859	1.25	0.087	
Pelargonic acid	0.760	0.772	0.558	0.740	0.926	0.933	0.697	0.866	1.24	0.146	
Creatine	122.976	123.220	138.281	154.257	160.225	150.556	128.159	155.013	1.21	0.010	**
Phosphorylcholine	5.483	4.118	3.323	4.277	5.197	6.017	4.308	5.164	1.20	0.348	
2-(Creatinine-3-yl)propionic acid	0.257	0.000	0.140	0.229	0.000	0.000	0.199	0.229	1.15	0.626	
Glucose 1-phosphate	4.894	6.519	8.025	5.571	5.895	10.851	6.479	7.439	1.15	0.646	
Lauric acid	2.003	1.745	1.621	2.038	2.296	1.772	1.789	2.036	1.14	0.262	
3-Hydroxybutyric acid	2.510	1.959	1.384	2.324	1.809	2.369	1.951	2.167	1.11	0.591	
Lactic acid	358.776	530.429	445.796	414.472	398.964	664.228	445.000	492.554	1.11	0.657	
Stachydrine	2.822	2.677	2.879	3.143	2.772	3.273	2.792	3.063	1.10	0.170	
S-Methylmethionine	0.000	0.000	0.076	0.068	0.084	0.092	0.076	0.081	1.08	0.098	
Terephthalic acid	0.949	0.826	0.729	0.790	0.909	0.971	0.834	0.890	1.07	0.541	
Trp	3.852	4.108	4.246	5.038	3.719	4.237	4.069	4.331	1.06	0.548	
S-Adenosylhomocysteine	0.051	0.048	0.085	0.073	0.059	0.064	0.062	0.065	1.06	0.780	
Urea	480.156	481.247	452.296	503.173	535.123	459.402	471.233	499.233	1.06	0.306	
N,N-Dimethylglycine	1.035	1.113	1.337	1.165	1.240	1.259	1.162	1.221	1.05	0.562	
Betaine	15.783	16.562	20.170	16.694	16.326	22.007	17.505	18.342	1.05	0.732	
Isobutyrylcarnitine	0.535	0.580	0.600	1.013	0.187	0.593	0.572	0.598	1.05	0.919	
Gly-Asp	0.413	0.415	0.397	0.363	0.449	0.463	0.408	0.425	1.04	0.635	
Taurine	329.803	332.411	325.795	344.740	357.554	324.828	329.336	342.374	1.04	0.251	
N-Acetylleucine	0.256	0.271	0.322	0.266	0.326	0.284	0.283	0.292	1.03	0.744	
His	21.605	22.718	22.006	21.559	23.144	23.471	22.110	22.725	1.03	0.413	
Asn	7.798	6.642	8.039	6.945	7.304	8.618	7.493	7.622	1.02	0.856	
Threonic acid	0.905	0.593	0.773	0.930	0.918	0.457	0.757	0.768	1.01	0.953	
1-Methylhistamine	0.112	0.059	0.086	0.107	0.081	0.070	0.086	0.086	1.01	0.980	
Octanoic acid	0.000	0.475	0.000	0.000	0.413	0.529	0.475	0.471	0.99	0.528	
UTP	7.006	7.296	7.568	9.282	6.162	6.089	7.290	7.178	0.98	0.921	
Homoserine	0.268	0.317	0.413	0.269	0.311	0.396	0.333	0.326	0.98	0.906	
5-Oxoproline	0.751	0.923	0.325	0.539	0.794	0.623	0.666	0.652	0.98	0.943	
Citrulline	17.041	15.130	24.090	19.232	17.724	17.712	18.754	18.223	0.97	0.857	
N6,N6,N6-Trimethyllysine	1.924	2.492	1.873	1.694	2.203	2.166	2.096	2.021	0.96	0.784	
Phe	24.025	21.590	23.109	24.959	18.181	22.798	22.908	21.979	0.96	0.684	
1-Methyladenosine	0.072	0.056	0.047	0.061	0.057	0.047	0.058	0.055	0.94	0.689	
Tyr	16.517	15.338	15.902	20.281	10.694	13.625	15.919	14.867	0.93	0.731	
Pipecolic acid	1.946	1.711	2.193	1.756	1.786	1.893	1.950	1.812	0.93	0.396	
2'-Deoxycytidine	0.111	0.139	0.080	0.116	0.083	0.105	0.110	0.102	0.93	0.700	
Glycerol	106.707	96.759	93.606	84.084	105.294	79.742	99.024	89.707	0.91	0.351	
Triethanolamine	0.703	0.290	0.436	0.511	0.497	0.275	0.476	0.428	0.90	0.751	
2-Hydroxyvaleric acid	0.404	0.463	0.450	0.397	0.524	0.220	0.439	0.380	0.87	0.548	
N5-Ethylglutamine	1.009	1.104	1.139	0.799	1.006	0.998	1.084	0.934	0.86	0.126	
Phosphoenolpyruvic acid	0.482	0.442	0.366	0.485	0.240	0.341	0.430	0.356	0.83	0.398	
Leu	45.426	44.190	53.109	41.947	37.406	38.385	47.575	39.246	0.82	0.055	
Thr	51.485	45.749	56.455	39.689	33.710	50.612	51.229	41.337	0.81	0.165	
Fructose 6-phosphate	24.289	35.756	40.495	22.896	20.735	37.114	33.513	26.915	0.80	0.402	
Glucose 6-phosphate	87.496	133.120	143.108	78.218	71.712	136.530	121.242	95.487	0.79	0.391	
Met	13.152	8.917	17.111	9.844	7.380	13.384	13.060	10.203	0.78	0.386	
1-Methylhistidine<br>3-Methylhistidine	3.133	2.435	2.489	2.174	2.124	1.863	2.686	2.054	0.76	0.061	
3-Phosphoglyceric acid	1.221	0.832	1.061	1.183	0.564	0.614	1.038	0.787	0.76	0.334	
Octanoylcarnitine	0.191	0.170	0.093	0.000	0.120	0.110	0.152	0.115	0.76	0.197	
CTP	1.394	1.751	1.641	1.526	1.028	1.035	1.595	1.196	0.75	0.111	
Ala	264.090	262.534	318.402	196.123	183.072	243.891	281.675	207.695	0.74	0.047	*
Ornithine	3.319	4.953	5.630	3.452	1.707	5.071	4.634	3.410	0.74	0.361	
Homocitrulline	0.424	0.614	0.492	0.306	0.423	0.385	0.510	0.371	0.73	0.101	
Methionine sulfoxide	0.864	0.920	0.971	0.731	0.367	0.872	0.918	0.656	0.72	0.164	
Isovalerylcarnitine	0.230	0.165	0.245	0.168	0.000	0.133	0.213	0.151	0.71	0.118	
Ser	34.916	34.585	38.225	24.870	23.124	26.913	35.909	24.969	0.70	0.002	**
Ile	27.235	25.019	29.412	19.373	19.087	18.075	27.222	18.845	0.69	0.003	**
Guanosine	0.000	0.136	0.000	0.099	0.000	0.089	0.136	0.094	0.69	0.768	
Val	56.254	52.804	62.643	38.407	33.731	41.750	57.233	37.963	0.66	0.006	**
ATP	452.141	436.120	454.260	420.963	238.829	223.685	447.507	294.493	0.66	0.074	
S-Lactoylglutathione	0.740	7.516	1.089	0.838	3.602	1.652	3.115	2.031	0.65	0.669	
2-Aminobutyric acid	1.650	1.466	2.087	0.993	0.962	1.068	1.734	1.008	0.58	0.018	*
Creatinine	8.397	8.154	7.400	5.196	3.958	4.172	7.984	4.442	0.56	0.002	**
PRPP	0.434	0.622	0.568	0.167	0.297	0.316	0.541	0.260	0.48	0.018	*
Urocanic acid	0.494	0.846	0.088	0.242	0.277	0.117	0.476	0.212	0.45	0.305	
Pro	62.195	57.735	90.002	26.716	19.520	38.728	69.977	28.321	0.40	0.023	*
Gly	230.867	249.833	247.630	61.317	82.751	62.558	242.777	68.875	0.28	0.000	***
Sarcosine	1.840	2.276	3.117	0.411	0.293	0.583	2.411	0.429	0.18	0.007	**
Phosphocreatine	224.953	214.487	207.952	99.273	4.440	8.198	215.797	37.304	0.17	0.005	**
β-Ala	9.625	7.125	13.230	1.722	1.131	1.620	9.993	1.491	0.15	0.009	**
Hydroxyproline	48.823	51.883	75.604	6.709	6.038	7.874	58.770	6.874	0.12	0.004	**
Anserine divalent	80.011	59.719	51.534	10.407	2.996	2.686	63.755	5.363	0.08	0.003	**
Homocarnosine	209.014	143.849	124.552	17.671	4.286	4.115	159.138	8.691	0.05	0.004	**
Carnosine	505.519	351.384	381.930	24.542	10.560	9.571	412.945	14.891	0.04	0.001	**
Pyruvic acid	0.000	4.225	0.000	0.000	0.000	0.000	4.225	0.000	not detected in Tg	0.374	
4-Methyl-2-oxovaleric acid<br>3-Methyl-2-oxovaleric acid	1.258	1.890	1.266	0.000	0.000	0.000	1.471	0.000	not detected in Tg	0.002	**
N-Methylalanine	0.394	0.366	0.262	0.000	0.000	0.000	0.341	0.000	not detected in Tg	0.001	**
6-Aminohexanoic acid	1.449	0.719	0.847	0.000	0.000	0.000	1.005	0.000	not detected in Tg	0.011	*
1-Methyl-4-imidazoleacetic acid	0.135	0.243	0.000	0.000	0.000	0.000	0.189	0.000	not detected in Tg	0.147	
N-Acetylserine	0.345	0.000	0.242	0.000	0.000	0.000	0.293	0.000	not detected in Tg	0.128	
β-Ala-Lys	0.229	0.281	0.286	0.000	0.000	0.000	0.265	0.000	not detected in Tg	0.000	***
Uridine	0.251	0.000	0.000	0.000	0.000	0.000	0.251	0.000	not detected in Tg	0.374	

S1 Table List of metabolites detected in CE-TOFMS

"Relative area" is the peak value of each metabolite normalized by sample volume; i.e., relative concentration of each metabolite. "Mean" is the mean value of the relative area from each group [WT and PGC-1 α -Tg (N = 3)]. "Ratio" is the comparative value of the relative areas (PGC-1 α -Tg per WT). "Not detected in WT" or "Not detected in Tg" means the peak of the metabolite was below the detection level in WT or Tg samples. P-value is calculated by Student's T-test (***$p < 0.001$, **$p < 0.01$, *$p < 0.05$).
