# Supplementary material for: Metabolomic Analysis of the Skeletal Muscle of Mice Overexpressing PGC-1α
Source: PLoS One. 2015 Jun 26;10(6):e0129084. doi: 10.1371/journal.pone.0129084 (PMC4482640; doi:10.1371/journal.pone.0129084)
Supplement: S2 Table — Fold changes of relative gene expression in skeletal muscle of each transgenic line compared with wild-type control mice are shown. Listed are genes used in the Figs 3–9. The data of PGC1α-b (B)/ PGC1α-b (03–2) are shown in the Figs 3–9. (PDF) [file pone.0129084.s003.pdf]

GeneName	Description	fold change vs WT		
		PGC1 α -a(E)	PGC1 α -b(A)/(02-1)	PGC1 α -b(B)/(03-2)
Csl	citrate synthase like (Csl)	1.6	2.1	2.6
Aco2	aconitase 2, mitochondrial (Aco2)	1.6	2.1	2.7
Idh2	isocitrate dehydrogenase 2 (NADP+), mitochondrial (Idh2)	1.2	1.5	2.8
Sdhb	succinate dehydrogenase complex, subunit B, iron sulfur (Ip) (Sdhb)	1.6	2.1	3.3
Mdh2	malate dehydrogenase 2, NAD (mitochondrial) (Mdh2)	1.3	1.8	2.3
G6pdx	glucose-6-phosphate dehydrogenase X-linked (G6pdx)	2.7	1.3	1.1
Ak3	adenylate kinase 3 (Ak3)	1.5	2.0	2.0
Adsl	adenylosuccinate lyase 1 (Adsl)	0.8	1.3	1.7
Ampd3	AMP deaminase 3 (Ampd3)	3.2	2.2	1.8
Slc25a11	solute carrier family 25 (mitochondrial carrier oxoglutarate carrier), member 11 (Slc25a11)	1.1	1.6	2.2
Slc25a13	solute carrier family 25 (mitochondrial carrier, adenine nucleotide translocator), member 13 (Slc25a13)	18.8	22.2	10.7
Bcat2	branched chain aminotransferase 2, mitochondrial (Bcat2)	1.2	1.7	2.5
Got1	glutamate oxaloacetate transaminase 1, soluble (Got1)	2.1	2.9	4.4
Got2	glutamate oxaloacetate transaminase 2, mitochondrial (Got2)	2.2	3.0	4.1
Mdh1	malate dehydrogenase 1, NAD (soluble) (Mdh1)	2.3	3.0	4.4
Mdh2	malate dehydrogenase 2, NAD (mitochondrial) (Mdh2)	1.3	1.8	2.3
Sdsl	serine dehydratase-like (Sdsl)	10.2	11.2	12.6
Gpt1	glutamic pyruvic transaminase 1, soluble (Gpt1)	2.8	3.1	3.6
Tha1	threonine aldolase 1 (Tha1)	1.4	2.0	2.1
Pdha1	pyruvate dehydrogenase E1 alpha 1 (Pdha1)	1.3	1.9	2.4
Pdhb	pyruvate dehydrogenase (lipoamide) beta (Pdhb)	1.5	2.0	2.5
Pdhx	pyruvate dehydrogenase complex, component X (Pdhx)	1.9	2.4	2.8
Shmt1	serine hydroxymethyl transferase 1 (soluble) (Shmt1)	1.0	1.9	2.3
Abat	4-aminobutyrate aminotransferase (Abat)	7.4	8.6	4.0

S2 Table List of gene expression change of transgenic lines of PGC1 α -b (B), PGC1 α -b (A)/ PGC1 α -b (02-1) and PGC1 α -b (B)/ PGC1 α -b (03-2) in microarray

Fold changes of relative gene expression in skeletal muscle of each transgenic line compared with wild-type control mice are shown. Listed are genes used in the Figures 3 to 9. The data of PGC1 α -b (B)/ PGC1 α -b (03-2) are shown in the Figures 3 to 9.
